# A remotely delivered exercise-based rehabilitation program for patients with persistent chemotherapy-induced peripheral neuropathy (EX-CIPN): Protocol for a phase I feasibility trial

**DOI:** 10.1371/journal.pone.0322371

**Published:** 2025-04-29

**Authors:** Eric M. Antonen, Michelle B. Nadler, David M. Langelier, Kristin L. Campbell, David Flamer, Jang Hyuk Cho, Scott Capozza, Lisa Avery, Kelcey A. Bland, Scott Leatherdale, Jackie Manthorne, Jennifer M. Jones

**Affiliations:** 1 Department of Supportive Care, Princess Margaret Cancer Centre, Toronto, Ontario, Canada; 2 Institute of Medical Sciences, University of Toronto, Toronto, Ontario, Canada; 3 Department of Medical Oncology & Hematology, Princess Margaret Cancer Centre, Toronto, Ontario, Canada; 4 Department of Oncology, Cumming School of Medicine, University of Calgary, Calgary, Alberta, Canada; 5 Department of Physical Therapy, University of British Columbia, Vancouver, British Columbia, Canada; 6 Anesthesiology & Pain Medicine, University of Toronto, Toronto, Ontario, Canada; 7 Department of Rehabilitation Medicine, Keimyung University Dongsan Hospital, Dongsan, Republic of Korea; 8 Rehabilitation Department, Yale New Haven Hospital, New Haven, Connecticut, United States of America; 9 School of Public Health Sciences, University of Waterloo, Waterloo, Ontario, Canada; 10 Canadian cancer survivor network, Ottawa, Ontario, Canada; PLOS: Public Library of Science, UNITED KINGDOM OF GREAT BRITAIN AND NORTHERN IRELAND

## Abstract

**Background:**

Chemotherapy-induced peripheral neurotoxicity (CIPN) is a prevalent adverse effect of chemotherapy agents that is estimated to be present in 2/3 of patients who receive neurotoxic chemotherapy. In 30–40% of these patients, CIPN signs and symptoms can persist for months or years post-treatment. Recent studies have supported exercise as a feasible and possibly effective intervention for CIPN; however, more rigorous studies are needed to confirm feasibility, estimate efficacy, and clarify risk. In response, we developed an innovative virtual exercise-based rehabilitation program (EX-CIPN) for cancer survivors with persistent CIPN.

**Methods:**

This study is a phase I study conducted at the Princess Margaret Cancer Centre in cancer survivors with persistent CIPN, with a focus on feasibility, acceptability, and safety. A total of 40 patients aged 18 or older, with persistent CIPN at least 6 months after chemotherapy completion will be recruited and receive the EX-CIPN program. The EX-CIPN program is a 10-week virtual home-based intervention that includes an individualized exercise program supported with a mobile application (Physitrack), wearable technology (FitBit), and weekly virtual check-ins with an oncology exercise specialist. The primary outcome of feasibility will be assessed by examining accrual, retention, and adherence rates. Acceptability will be assessed through qualitative interviews. Safety events will be monitored and reported based on CTCAE v5. Secondary outcomes will be collected using questionnaires and physiological assessments at baseline (T1), after the intervention (T2), and 3-months after intervention (T3).

**Conclusion:**

This phase I study will determine intervention feasibility, acceptability, and safety and will inform the planning for a future Phase II RCT with the EX-CIPN intervention.

## Background

Improvements in cancer detection and treatment have led to an increase in the prevalence of people living with a personal history of cancer [1]. As a result, the long-term effects of cancer and its treatments on chronic morbidity and disability are of increasing importance [2, 3].

Chemotherapy is a cornerstone therapy used to treat many common cancers and prevent their recurrence; however, it can result in persistent toxic effects [4]. Chemotherapy-induced peripheral neurotoxicity (CIPN) is a prevalent adverse effect of chemotherapy agents [5–7] which can develop during chemotherapy receipt or after treatment completion [8]. It is estimated the 2/3 of patients who receive neurotoxic chemotherapy will develop CIPN [9]. Acute CIPN develops during treatment and generally improves over the first 3–6 months following treatment completion [9,10]; however, in 30–40% of patients (higher for those treated with taxanes and platinums), CIPN symptoms can persist for months or years post-treatment [9,11–14].

While the pathogenesis of CIPN is not completely understood, CIPN is a predominately sensory axonal neuropathy that typically affects distal limbs in a length dependent pattern [15]. The sensory involvement of CIPN can result in pain, numbness, paresthesia, temperature sensitivity, and/or proprioception loss [16]. Sensory loss can lead to static and dynamic instability, gait disturbances, and increased appendicular muscle weakness from disuse, resulting in physical deconditioning and an increased risk of falls. The risk of falls in cancer patients with CIPN is almost double compared to those without it [17]. Persistent CIPN also has a profound impact on overall quality of life and can result in compromised social well-being and act as a barrier to returning to work after cancer treatments are completed [18–23].

Recent systematic reviews suggest treatment options for symptoms of persistent CIPN are of limited or uncertain benefit [24, 25]. Pharmacological agents that are effective for the treatment of similar axonal, length dependent diabetic and HIV-related neuropathies, such as tricyclic antidepressants and antiepileptic drugs (gabapentin and pregabalin) do not improve CIPN [24,26–28]. Duloxetine is currently the only recommended pharmacological agent for the symptomatic treatment of CIPN [29], though its benefit is limited and it has not been studied broadly across neuropathy causing therapies [29]. Further, duloxetine can be contraindicated in those receiving tamoxifen (a common endocrine therapy for breast cancer) [30], amongst patients receiving other mood stabilization medications [31], and requires caution when used for older adults with cancer [32]. Given the uncertain benefits and known side effects of pharmacologic therapies along with cancer survivors’ desire to limit any further required medication [33], research examining a wider range of non-pharmacologic interventions including lifestyle interventions, such as exercise has emerged, thus supporting this study [34].

Exercise-based rehabilitative interventions are effective for managing many side effects of cancer treatment and enhancing functional abilities in individuals affected by cancer [35, 36]. Exercise is feasible and may be an effective intervention for different forms of peripheral neuropathy [37, 38] and for patients experiencing CIPN [39–41] and observational studies have demonstrated connections between higher levels of physical activity and milder cases of CIPN [20,42]. There are several neurophysiological mechanisms through which exercise may potentially alleviate peripheral neuropathy, including the induction of an anti-inflammatory environment, increasing the supply of blood, glucose and oxygen to mitochondria, and by affecting psychosocial processes [43–46]. To date, while the evidence on exercise for symptomatic treatment of CIPN demonstrates encouraging positive effects on CIPN-related outcomes [39–41], it is limited by low study quality and a lack of definitive rigorous trials with CIPN symptoms as the primary end-point [38,40,41]. In its most recently updated guideline, the American Society of Clinical Oncology (ASCO) state that “preliminary supportive evidence” exists in favor of exercise to treat CIPN, but concluded that “no recommendation can be made” due to the lack of robust evidence and recommends that more research is needed to confirm efficacy and clarify risks [29].

Traditional in-person supervised exercise-based rehabilitation interventions for people with cancer have been facility-based (e.g., hospital or university), where people received exercise counselling from a trained professional or completed an exercise session under direct supervision of a trained exercise professional. However, recently supervised, live remote exercise interventions [47] have been shown to be safe, feasible, and effective [48–50]. Virtual delivery can support remote delivery of exercise guidance or live remote supervision of an exercise session. Virtual interventions arose in response to patient barriers to attending facility-based programming, including the lack of locally available cancer-exercise programs, inflexible program hours, costs, transportation issues, and symptom burden [51–54]. Most people with cancer report a preference for exercising at home, if provided with appropriate guidance from a qualified oncology exercise professional [53–55] and evidence demonstrates good compliance and positive experiences with remote platforms for exercise delivery [56]. Most exercise studies for the symptomatic treatment of CIPN have been delivered in supervised, in-person settings [57]. There are no prior studies of any virtually delivered exercise-based rehabilitation interventions for the treatment of CIPN. Further, the incorporation of behaviour change theory and behaviour change techniques is recommended in the development of interventions that require patient’s to adopt health-related behaviours [58]; however, a recent review of exercise interventions for the prevention and management of CIPN found that no study has incorporated a clear theoretical or conceptual framework related to behaviour change [59].

In response, we developed an innovative remotely delivered 10-week exercise-based rehabilitation program (EX-CIPN) for cancer survivors with persistent CIPN. The proposed study is of scientific interest and clinical importance because: (a) neurotoxic chemotherapy remains a cornerstone of cancer therapy; (b) CIPN is a common persistent side effect that severely affects quality of life and function of the increasing number of cancer survivors in Canada; (c) there are currently few strongly supported symptomatic treatment options available for CIPN; and (d) virtual exercise-based rehabilitation is a promising and accessible intervention for CIPN

## Methods

**Fig 1:**
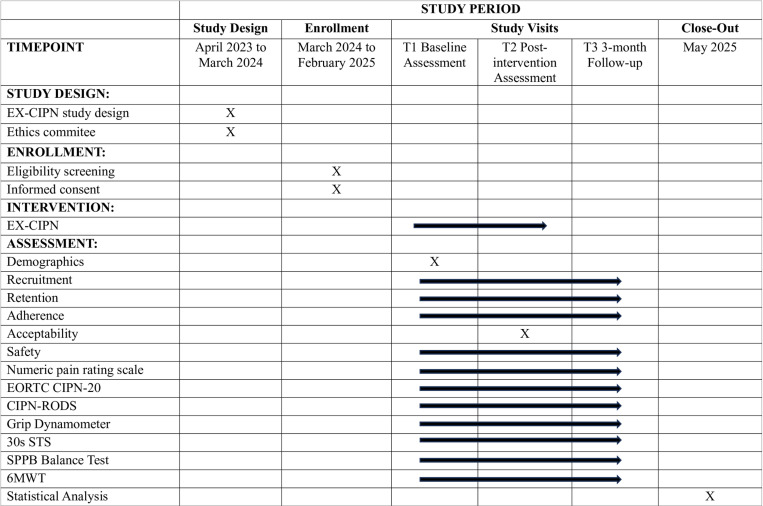
SPIRIT statement.

**Fig 2:**
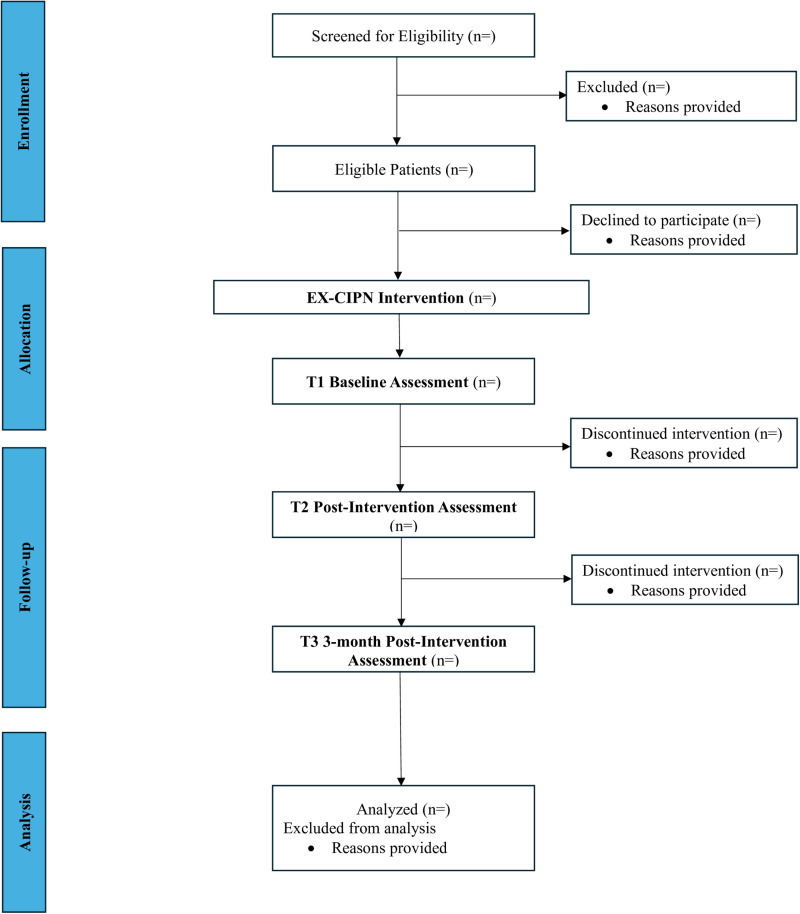
CONSORT participant flow diagram.

**Fig 3:**
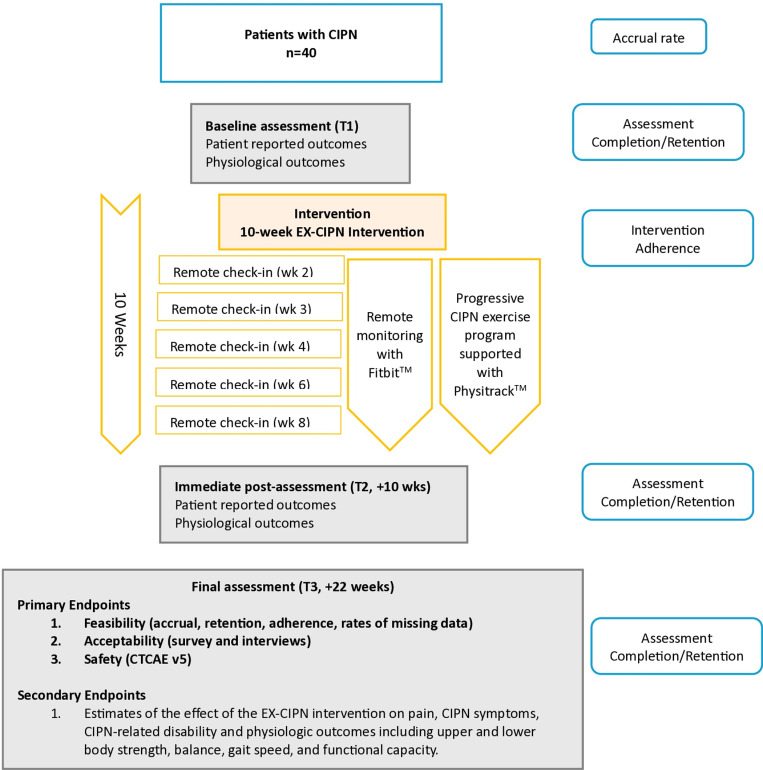
Study flow diagram.

The proposed study is a Phase I trial of the EX-CIPN intervention with patients experiencing persistent CIPN. The phase I trial is a multi-method, single-group, pilot study. This Phase I pilot study is important to conduct before a Phase II RCT as it allows us to determine if the program we have developed is safe, practical, and satisfactory for our patient population before we perform a larger confirmatory study. The study protocol is reported according to the Consolidated Standards of Reporting Trials (CONSORT) 2010: extension to randomized pilot and feasibility trials and Standard Protocol Items: Recommendations for Interventional Trials (SPIRIT)[60, 61] (see Fig 1). The CONSORT [61] participant flow diagram can be found in Fig 2. Following enrollment in the study, all participants will complete the baseline assessment (T1) and will then take part in the 10-week EX-CIPN program. They will complete follow-up assessments immediately post-intervention (T2) and 3-months post-intervention (T3) (see study flow Fig 3). This trial has been registered with clinical trials.gov (NCT06405542) and has been approved by the University Health Network Research Ethics Board (REB# 23–5839). The trial is currently still actively recruiting and collecting data. Recruitment is expected to be completed in April 2025 and data collection in September 2025. Based on this timeline completion of data analysis and results are expected in October 2025.

### Study population and recruitment

Potential participants will be recruited from Princess Margaret Cancer Centre from outpatient oncology clinics and the study will be promoted on Princess Margaret social media channels and through study flyers. These flyers will be distributed in clinic waiting areas at Princess Margaret and other hospitals in the Greater Toronto Area.

All potential participants will undergo a screening process to determine eligibility. Individuals who meet the study criteria and provide written consent to the study coordinator will be scheduled to complete an in-person baseline physiological assessment (T1) at the ELLICSR: Cancer Rehabilitation and Survivorship Centre. In addition, they will receive an electronic link via Research Electronic Data Capture tools (REDCap) to a set of questionnaires to assess patient reported outcome measures prior to their baseline assessment (T1). Follow-up assessments will be conducted at 10 weeks (T2) (immediate post-intervention) and 22 weeks (T3) (3-month post intervention). Feedback to assess acceptability will be collected at the end of the study though a participant satisfaction survey and qualitative interviews (*see* Fig 3 *for study schema*).

Participants will be eligible if they: a) are ≥ 18years of age; b) were diagnosed with cancer and treated with curative intent c) received chemotherapy as part of their curative intent treatment (no minimum dose) d) are >6 months following chemotherapy completion (i.e., no other chemotherapeutic agents since completion of the chemotherapy regimen) with no current plans for chemotherapy in the next 6 months; e) report ≥ Grade 1 on the numbness and tingling severity item of the NCI Common Terminology Criteria for Adverse Events (CTCAE) v 5.0[62] meaning mild to severe symptoms and neuropathic pain ≥3 on the Neuropathic Pain 4 (DN4) (interview) (0–7); f) currently engaging in <90 minutes per week of moderate-intensity aerobic exercise; g) independent with ambulation and transfers with or without ambulatory assistance (Eastern Cooperative Oncology Group (ECOG) score 0–2); h) Able to communicate sufficiently in English to complete intervention, questionnaires, and consent; i) Have access to and are able to operate videoconferencing.

Participants may be on maintenance oncologic therapies (i.e., endocrine therapy, PARP inhibitors) not known to cause neuropathy. In addition, they may be on oral or topical medications, or local-regional therapies to treat neuropathy at the start of the study, if the doses or modalities are unchanged over the past 6 weeks and symptoms of neuropathy persist. Participants will be asked to not change the dose of their neuropathy medications, cream, or complementary therapies, or start a new treatment for neuropathy during the course of the study. Medication and complementary therapy use will be tracked.

Additional exclusion criteria include: a) neurological conditions such as dementia and Alzhemier’s influencing cognition and preventing safe or appropriate engagement with exercise recommendations; b) pre-existing neuropathy or neuromuscular disorders prior to chemotherapy; c) current enrollment in other rehabilitation or exercise-based interventions.

### EX-CIPN intervention

EX-CIPN is a 10-week remotely delivered home-based program developed from previous evidence and guidance regarding CIPN and exercise [41,63–68], exercise guidelines for cancer survivors, and established behaviour change theory and techniques to promote uptake of behaviours [69–74]. The remote delivery of EX-CIPN helps to address barriers to accessing and providing rehabilitation and provides a cost-effective model that can be widely adopted. EX-CIPN is comprised of the following: (1) a progressive 10-week exercise program (aerobic, resistance and balance) supported with a mobile application (Physitrack^®^) and wearable technology (Fitbit™) to track activity; and (2) brief video/telephone check-ins provided by a Registered Kinesiologist (RKin) who is trained in motivational interviewing. Grounded in behavior change theory, this program equips participants with the knowledge and tools necessary to achieve and sustain their exercise goals. It integrates multiple theoretical frameworks, including motivational interviewing, cognitive behavioral therapy, and the transtheoretical model of behavior change. The intervention focuses on resolving motivational hesitancy, identifying and modifying cognitive distortions that hinder the adoption of healthy behaviors, and long-term behavior maintenance [67–72]. Participants receive the program at no cost, except for personal expenses such as public transportation or parking for in-hospital assessments.

Informed by behavior change theory, the program components provide participants with the knowledge and tools needed to reach and maintain their exercise goals. Multiple theoretical models are integrated within the intervention (i.e., motivational interviewing, cognitive behavioral therapy, transtheoretical model of behaviour change), with the focus on addressing and resolving motivational ambivalence and identification and modification of the cognitive distortions that prevent adoption of appropriate health behaviors and addresses relapse and long-term maintenance of behavior change [69–74]. The program is provided free-of-charge to participants, other than out of pocket travel costs (i.e., public transport, parking) for in-hospital assessments.

#### Individualized progressive exercise program.

**Fig 4:**
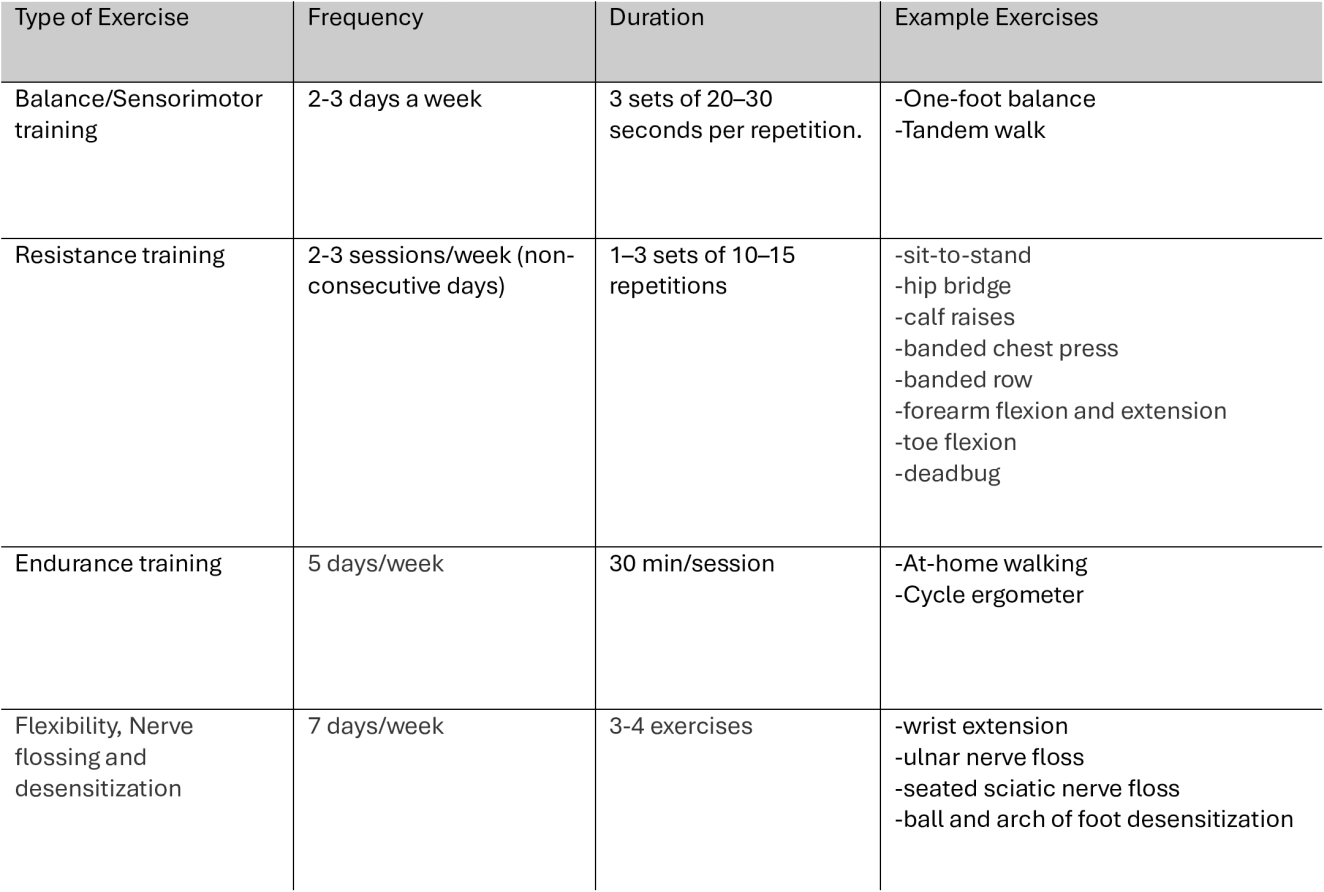
Sample exercise program.

Each participant will receive an individualized progressive multi-modal exercise program based on current exercise guidelines for CIPN and recent published evidence [41,66,75,76]. All exercise targets will be adjusted to the individual needs of the participant and baseline physical fitness levels. The program will aim to reach at least 150 minutes of moderate intensity aerobic exercise per week (e.g., 30 minutes of exercise 5 days per week), and resistance and balance exercises will be prescribed 2–3 times per week. Flexibility and nerve desensitization will be prescribed daily (Fig 4). The types of aerobic exercise that will be recommend to participants include brisk walking and cycling. Resistance training will include exercises that target major upper and lower body muscles groups, such as sit-to-stand, chest press, and back rows using body weight and elastic bands. Flexibility exercises used such as calf and wrist extension stretches and nerve gliding/flossing exercises will include sciatic and ulnar nerve flossing to target common areas known to be affected by CIPN. Finally, desensitization exercises including ball of foot and arch of foot exercises with a textured ball have been shown in previous literature to improve CIPN symptoms such as numbness and loss of sensation [75]. The exercise program will be revised and progressed during scheduled check-ins through the prescription of more advanced exercises and an increased exercise intensity by increasing repetitions and sets. The exercise program will be supported by Physitrack^®^. Physitrack^®^ is an online application that allows RKins to remotely edit, and progress participants exercise programs and allows participants to view videos and descriptions of their prescribed exercises. The participants will be introduced and oriented to the Physitrack^®^ software at the T1 assessment.

#### Remote monitoring.

To remotely monitor step count and physical activity participants will be provided Fitbit^™^ devices for the duration of the program. To track this data and speak about it at check-in calls, the designated oncology exercise specialist will have access to Fitbit^TM^ data. Fitness trackers can enable self-monitoring and feedback to participants, ultimately promoting behaviour change [77]. Instructions are provided for Fitbit^™^ device and application set-up. Any additional support requested will be provided by the assigned RKin.

#### Remote person-to-person clinical support.

Participants will have scheduled remote health coaching check-ins over MS Teams video call or telephone if needed. This person-to-person component can foster accountability and facilitate social support and tailored feedback for participants from their assigned RKin [76, 77]. Check-ins will be scheduled on weeks 2, 3, 4, 6, and 8 of the program. During these calls, the RKin will discuss program progression with the participant and adapt or progress the exercise program as needed throughout the intervention. The participant will also be guided to speak on any changes they have experienced since the last check-in call or any barriers to the program that have arisen. Based on this feedback participants will discuss and develop goals as well as solutions to their barriers. The RKin will also go through a CTCAE v5.0 checklist with the participant at each support call to keep track of any adverse events. The RKins facilitating the intervention have received training in motivational interviewing (MI) from a certified Motivational Interviewing Network Trainer. They will integrate strategies to assess and enhance intrinsic motivation, foster self-efficacy, and apply a collaborative problem-solving approach [69,78]. MI is designed to encourage and sustain behavioral change by addressing ambivalence, and it has been demonstrated to effectively increase physical activity in populationss with chronic conditions, including cancer [69,79,80].

## Outcomes

### Primary outcomes

*To determine if EX-CIPN is feasible (accrual, retention, and adherence), acceptable, and safe in patients experiencing persistent CIPN.*

#### Feasibility.

- Recruitment and accrual will be tracked based on CONSORT criteria [61] through a screening log that tracks all referred and screened patients. Eligibility screening will also be used. This screening will find eligible and consenting participants as well as eligible non-recruited patients with reasons documented. *Target: accrual of four participants per month.*
- The percentage of study participants that attend T1, T2, and T3 assessments will be used to assess retention rates. Rates of complete and missing data will also be measured. *Target: retention of ≥70% at each study time-point.*
- Adherence to the intervention will be assessed through check-in call attendance, Fitbit™ usage, and self-report completion of weekly exercise plan (determined during weekly calls). *Target: adherence of ≥70% to each of the intervention components.*

#### Acceptability.

- All participants will be asked to complete a brief satisfaction survey during their T2 assessment. In addition, we will conduct in-depth, semi-structured, qualitative, one-on-one video interviews with 10-12 participants following the T3 assessment. This is the number anticipated to support data adequacy in terms of the amount and variety of evidence that will be collected. Thematic analysis will be conducted [81] and an interpretive descriptive qualitative methodology will be used [62,82–85]. *Target: high levels of treatment acceptability based on satisfaction survey (≥ 75%) and interview data.*

#### Safety.

- All adverse events will be scored on the CTCAE 5.0 [62] and documented during check in appointments and at follow-up assessments. *Target: <10% of participants experience serious adverse events (>Grade 3 of the CTCAE 5) directly related to the study or intervention.*

### Secondary outcomes

Patient-reported outcomes measures (PROMs) and physiologic assessments will be completed at each time point. PROMs include the numeric pain rating scale (pain)[86], the EORTC CIPN-20 (CIPN symptoms)[87], and CIPN-RODS (CIPN-related disability)[88]. Physiologic outcomes include upper (grip dynamometer) and lower body strength (30-second sit-to-stand test), balance (Berg Balance Test), gait speed (4-metre gait speed test), and functional capacity (6-minute walk test).

### Sample Size

Although there is no definitive consensus on the ideal sample size for a feasibility study [89], simulations of various sample sizes and standard deviation values for precision estimation (α = 0.05, power = 80%) indicate that 35–40 falls at the elbow point of the curves. Consequently, a sample size of 40 participants has been selected, which is considered sufficient to assess the feasibility of the study [90, 91].

### Data Analysis

A database with participant details and study progress, only available to authorised study staff, will be kept in encrypted and protected files on University Health Network servers to be stored securely. All data from the secondary outcomes will be stored on REDCap, which is an application developed to capture data for clinical research that provides a secure method for data collection and storage. Data audits, restricted data access, and consistent data monitoring will be used to maintain data quality and integrity.

### Primary Outcomes

Participant baseline characteristics and study feasibility will be reported using descriptive statistics. Study feasibility will include: 1) accrual rate (average number of patients per month) 2) the percentage and total number of patients who are eligible with ineligibility reasons documented; 3) the total number of consented participants with declined consent reasons documented; and, 4) attrition rates at each assessment with drop-out reasons documented. The feasibility of the intervention will be assessed by examining: 1) Fitbit^TM^ usage; 2) Weekly virtual check-in attendance; and 3) Patient self-report completion of weekly exercise plan.

Acceptability will be evaluated using qualitative interview data, followed by thematic analysis.[92]. The analysis will primarily follow a deductive approach, with predefined categories designed to align with the core program components and capture participants’ experiences, including perceived benefits, limitations, and areas for improvement. After the initial coding, interviews will be reviewed again to identify any additional themes through an inductive process. Themes will be developed by closely examining the codes and categories, exploring their relationships, and engaging in discussions with the research team [93].

Any program-related safety event will be documented according to the Common Terminology Criteria for Adverse Events v5.0[62]. Safety events will be classified according to their presumed connection to the intervention (e.g., definitely related, possibly related, or definitely unrelated).

### Secondary Outcomes

Capture rates of the patient-reported outcomes will be assessed and described at each time point to inform future sample size calculations. This analysis is not powered, and the goal of the analysis is to estimate the effect size and 95% confidence intervals from baseline to follow-up. Data analysis will commence upon the completion of recruitment and data collection. The program used to perform the analysis is RStudio version 4.0.4 (R Project for Statistical Computing).

### Interpretation of Results

Our intervention will be considered feasible for a Phase II RCT if it achieves a consistent recruitment rate of 4–5 participants per month [43,93], maintains a retention rate of at least 70% at each study time point [43,64], and demonstrates satisfactory adherence (70%) to key intervention components, including Fitbit usage, participation in scheduled calls, and completion of weekly exercise plans [43]. Also, intervention success will be measured using acceptability of ≥ 75% on post-study surveys and high levels of acceptability from qualitative interview data. The intervention will be considered safe if no serious adverse events occur, defined as any event above Grade 3 according to the CTCAE v5, related to participation in the study. If any of these criteria are not fulfilled, we will adjust the protocol accordingly before proceeding with a larger RCT. Calculation of the sample size for a Phase II RCT will be based on a minimally important clinical difference of 2 points between the T1 and T3 on the numeric pain rating scale [86].

## Discussion

The use of an exercise-based rehabilitation program with a remote delivery strategy in the standard treatment for patients with CIPN has the potential to improve patients’ symptoms of neuropathy, improve disability, and improve physical functioning [39–41,43]. However, rehabilitation programs are not typically available for the specific needs of those with CIPN as the current research is limited. The proposed study aims to address current gaps in the existing evidence. The EX-CIPN study uses an exercise-based approach with behaviour change strategies to target physical activity and behaviour change to support improved function [41,43,69–72]. The primary objective of this study is to assess the feasibility, acceptability, and safety of the EX-CIPN program. The outcomes of this pilot will yield fundamental data that will guide the creation of a Phase II RCT.

### Strengths and Limitations

The EX-CIPN intervention itself has many strengths as it is based on the current literature and experiences through similar programs [39–41,43,94] and is developed using different behaviour change theories and techniques built into the design [69,73,74]. The remote nature of the intervention removes many barriers to accessing and providing rehabilitation and allows for a cost-effective model that can be widely adopted [52–56]. The program uses many tools further supporting behaviour change such as wearable technology and person-to-person remote check-in calls that also add to the ease of program completion through their remote nature [78]. Finally, the quality of the study methods has been enhanced through the use of the SPIRIT 2013 Statement (Standard Protocol Items: Recommendations for Interventional Trials) during development [60, 61].

This study design, however, does have limitations. We understand that the remote nature of the program is meant to add to its ease of use and accessibility, however, with the inclusion of in-person assessments conducted in one specific tertiary care centre, located in an urban setting, this may limit the study’s accessibility and generalizability to more rural communities. Further, due to the lack of existing evidence in the field it will not be possible to conduct this as a randomized controlled trial. Therefore, as this is a phase I trial, we will be unable to conclude if any improvements that may be detected are due to the intervention itself. Finally, as patients with pre-existing neuropathy are excluded this may exclude some patients who have developed further neuropathy from chemotherapy. To improve the program and research design and help with Phase II RCT preparation, all expected and unexpected difficulties will be highlighted, and solutions will be recorded.

## Conclusion

This study addresses the need for individualised, remote, exercise-based rehabilitation programs for patients with persistent CIPN. The potential clinical implications are considerable, offering improved outcomes and symptoms for patients and informing exercise-based programs. The study’s strengths lie in its remote nature, use of pre-existing clinical experience, and targeted patient population. The findings from this study have the potential to provide substantial contributions to the field of exercise-based rehabilitation and treatment of persistent CIPN.

## Supporting information

S1 Checklist SPIRIT 2013 Checklist. (DOC)

S1 Protocol EX-CIPN_Protocol_Jan23_2024_Clean. (DOCX)
