# Supplementary material for: A remotely delivered exercise-based rehabilitation program for patients with persistent chemotherapy-induced peripheral neuropathy (EX-CIPN): Protocol for a phase I feasibility trial
Source: PLoS One. 2025 Apr 29;20(4):e0322371. doi: 10.1371/journal.pone.0322371 (PMC12040081; doi:10.1371/journal.pone.0322371)
Supplement: S1 Protocol — (DOCX) [file pone.0322371.s002.docx]

**CLINICAL TRIAL PROTOCOL**

**EX-CIPN: A Phase II Trial of an exercise-based rehabilitation intervention to treat persistent Chemotherapy-Induced Peripheral Neuropathy (CIPN)**

**Principal Investigator:**

Jennifer M Jones^1^

**Co-Principal Investigator:**

Michelle Nadler^2^

**Co-Investigators:**

David Langelier^1^

Kristin Campbell^3^

David Flamer^4^

Jang Hyuk Cho^5^

Scott Capozza^6^

Lisa Avery^7^

Kelcey Bland^3^

*Jackie Manthorne^8^

*Scott Leatherdale^9^

^1^ Cancer Rehab and Survivorship (CRS) Program, Princess Margaret Cancer Centre

^2^ Division of Medical Oncology & Hematology (DMOH), Department of Medicine; Princess Margaret Cancer Centre

^3^ Department of Physical Therapy, University of British Columbia

^4^ Division of Pain Medicine | Sinai Health, University Health Network, Women's College Hospital

^5^ Department of Rehabilitation Medicine, Keimyung University School of Medicine

^6^ Smilow Cancer Hospital at Yale New Haven Health/Rehabilitation Department, Yale New Haven Health

^7^ Department of Biostatistics, Princess Margaret Cancer Centre

^8^Canadian Cancer Survivor Network (CCSN)

^9^ School of Public Health Sciences, University of Waterloo

******Patient partner*

**Study Sponsor:** Principal Investigators

**Source of Funding:** Pending

**Coordinating Centre**: Supportive Care Clinical Trials Unit, Princess Margaret Cancer Centre

**PI:** Jennifer M Jones, PhD

Cancer Rehabilitation and Survivorship Program, Princess Margaret Cancer Centre

University Health Network, 200 Elizabeth Street, PMB-B-045

Phone: 416-581-8603 (office)/416-786-4583 (cell)/ Email: jennifer.jones@uhn.ca

**Table of Contents**

**SECTION PAGE**

List of abbreviations/terminology 3

Protocol Synopsis 4

Study Schema (Figure 1) 8

Statement of compliance 9

1.0 Objectives 10

2.0 Background and Rationale 10

3.0 Experimental Design and Methods 17

4.0 Study Intervention(s)/Procedure(s) 20

5.0 Study Outcome/Endpoints 22

6.0 Sample Size and Timeline 24

7.0 Data Management 24

8.0 Analyses 25

9.0 Criteria for positive trial/interpretation of results 26

10.0 Data collection, handling, and storage 26

11.0 Ethics 28

12.0 Direct Access to Source Data/documents 29

13.0 Publication and Data Availability Policy 30

14.0 Time to Study Initiation and Milestone Achievements 30

References 32

Tables and Figures

# **List of Abbreviations/Terminology**

ACSM American College of Sports Medicine

ANCOVA              Analysis of covariance

ASCO American Society of Clinical Oncology

CIPN Chemotherapy induced peripheral neuropathy

CIPN-RODS Chemotherapy induced peripheral neuropathy-Rasch built Overall Disability Scale

CONSORT Consolidated Standards of Reporting Trials

CTCAE                  Common Terminology Criteria for Adverse Events

CTL Control

EORTC European Organization for Research and Treatment OF Cancer

EORTC-QLQC30 EORTC core quality of life questionnaire

ECOG Eastern Cooperative Oncology Group

ESMO European Society for Medical Oncology

EX-CIPN Exercise-based rehabilitation program

INT Intervention group

MI Motivational interviewing

MS Teams           Microsoft Teams

NCI National Cancer Institute

QoL                       Quality of life

RCT Randomized controlled trial

REB                       Research ethics board

RedCap Research electronic data capture tool

RKin Registered kinesiologist

SPIRIT Standard protocol item: recommendations for intervention trials

UC Usual care

**PROTOCOL SYNOPSIS**

| **Protocol Title** | |
| --- | --- |
| Ex-CIPN: A Phase II Trial of an exercise-based rehabilitation intervention to treat Chemotherapy Induced Peripheral Neuropathy (CIPN) | |
| **Study Detail** | |
| **Study Population/Indication (s):** | All cancers; within 6-12 months of completion of taxane or platinum-based chemotherapy; reporting persistent CIPN |
| **Study Design:** | Single-centre single-group Phase I trial |
| **Sample Size:** | 40 |
| **Estimated Accrual Duration:** | 15 months |
| **Estimated Study Duration:** | 24 months |
| **Participating Sites:** | Princess Margaret Cancer Centre, Toronto, ON, CANADA |
| **Intervention:** | EX-CIPN, a tailored progressive remotely delivered 10-week exercised based rehabilitation intervention. |

**Rationale for Study**

Improvements in early detection and the treatment of cancer have led to a substantial increase in the number of people living with a personal history of cancer. With increasing prevalence of people now surviving cancer and transitioning into the extended survival phases of cancer care, the long-term effects of cancer and its treatments and previously unrecognized chronic morbidity and related disability are of increasing importance.

Chemotherapy-induced peripheral neurotoxicity (CIPN) is a prevalent adverse effect of chemotherapy agents that is estimated to be present in 2/3 of patients who receive neurotoxic chemotherapy. In 30-40% of patients (higher for those treated with taxanes and platinum analogues), CIPN signs and symptoms such as neuropathic pain or decreased sensation can persist for months or years post-treatment. CIPN has a profound impact on the quality of life for individuals who have undergone treatment for cancer, including pain, weakness, physical disability and impairment, falls, compromised social well-being, and obstacles in returning to work after treatments are completed. Currently, there are no evidence-based approaches for preventing CIPN and symptomatic treatment options for established CIPN are limited and of uncertain benefit.

Recent evidence has shown that exercise is feasible and may be an effective intervention for different forms of peripheral neuropathy and with patients experiencing CIPN. However, professional organizations such as the American Society of Clinical Oncology (ASCO) and the European Society for Medical Oncology (ESMO) have not made any strong recommendations on the use of exercise-based interventions for the prevention or symptomatic treatment of CIPN. In its most recently updated guideline, ASCO stated that “preliminary supportive evidence” exists in favor of exercise to prevent and treat CIPN, but concluded that “no recommendation can be made” due to the lack of robust evidence and recommends that more rigorous definitive studies are needed to confirm efficacy and clarify risks. Further, there has been no study of a remotely delivered theory-based exercise-based rehabilitation intervention for the symptomatic treatment of persistent CIPN.

In response, we developed an innovative remotely delivered 10-week exercise-based rehabilitation program (EX-CIPN) for cancer survivors with persistent CIPN. As a first step, we propose a Phase I study to explore the feasibility and acceptability of the program and methods, rigorously track safety, and describe the preliminary effects of the program on clinical outcomes.

**Objectives**

***Primary Objectives***

Primary Objective 1: *To determine if EX-CIPN is feasible, acceptable and safe in cancer survivors experiencing persistent CIPN.*

Primary Objective 2: *To examine the feasibility of the study design and methods (recruitment and retention rates, feasibility of data collection and procedures).*

***Secondary Objective***

To obtain estimates of the effects of EX-CIPN on pain (primary clinical outcome), CIPN symptoms, CIPN-related disability and physiologic outcomes including upper and lower body strength, balance, gait speed, and functional capacity (secondary exploratory outcomes).

**Study Design**

The proposed study is a single centre single-group Phase I trial with patients who are experiencing persistent CIPN. All participants will receive the EX-CIPN intervention. EX-CIPN is comprised of: a progressive 10-week exercise program (aerobic, resistance and balance) supported with a mobile application (Physitrack^®^) and wearable technology (Fitbit™) to track activity; and (2) weekly brief video/telephone check-ins provided by an oncology exercise specialist who is trained in motivational interviewing. Informed by behavior change theory, the program components aim to provide patients with the knowledge and tools needed to reach and maintain their wellness and exercise goals. The remote delivery of EX-CIPN helps to barriers to accessing and providing rehabilitation and provides a cost-effective model that can be widely adopted. There will be three assessment time points throughout the study (T1, T2, T3) all including patient reported outcomes and a physiologic assessment. T1 is a baseline assessment before the intervention begins, T2 is a post-intervention assessment, and T3 is a 3-month follow-up after intervention completion.

**Study Endpoints**

The objectives will be measured by the following corresponding endpoints.

***Primary Endpoints:***

**Primary Endpoint 1: *To determine if EX-CIPN is feasible (retention and adherence), acceptable, and safe in cancer survivors experiencing persistent CIPN.***

**Primary Endpoint 2: *To examine the feasibility of the study design and methods (accrual and retention rates, feasibility of data collection and procedures).***

- Recruitment and accrual will be tracked based on CONSORT criteria^93^ through a screening log that tracks data collected from all referred and screened patients. Eligibility screening will be performed to identify eligible, consented, and eligible non-recruited individuals with non-recruitment reasons documented.

*We will define our intervention as feasible if we are able to accrue an average of five (5) participants per month.*

- Retention rates at each study time point will be monitored. Retention rates will be calculated as the proportion of recruited participants that attend each assessment time point (T1, T2, and T3). We will also examine rates of complete and missing data.

*We will define our intervention as feasible to test in a subsequent RCT if there is reasonable retention (>70%) at each study time-point, in both arms****.***

- Adherence to the intervention will be assessed through health coaching call attendance, Fitbit™ usage, and self-report completion of weekly exercise plan (determined during weekly calls).

*We will define our intervention as feasible to test in an RCT if there is reasonable adherence (>70%) to the intervention components (including Fitbit usage, call attendance, and completion of weekly exercise plans).*

- Safety will be assessed throughout the study. All adverse events will be scored on the CTCAE version 5.0 ^9^ and documented during weekly appointments and at follow-up assessments with the RKin.

*Safety of the intervention will be confirmed if <10% of participants experience serious adverse events (defined as anything above a Grade 2 of the CTCAE v5) related to participation in the study or intervention occur.*

- To evaluate acceptability and guide future program refinement, we will ask all participants to complete a post-study survey immediately after T2 to provide feedback on their experience. We will also conduct in-depth semi-structured qualitative video interviews (MS Teams) with a sub-sample of approximately 1/3 of participants following the T2 assessment (including those who improved in CIPN symptoms and those who did not improve).

*Success will be measured by high levels of treatment acceptability based on qualitative survey and interview data.*

***Secondary Endpoints***

**Secondary Endpoint 1: Reduction in the patient-reported CIPN-related pain (primary clinical outcome) will be summarized in the intervention and control groups.**

*Calculation of the sample size for a larger RCT will be based on distribution of change observed in this pilot and a minimally important clinical change of 2 points on the numeric pain rating scale from T1-T3 assessment.*

Secondary Endpoint 2: Estimates of the effect of the EX-CIPN intervention on CIPN symptoms, CIPN-related disability and physiologic outcomes including upper and lower body strength, balance, gait speed, and functional capacity (exploratory secondary outcomes).

**Abbreviated Eligibility Criteria**

***Main Inclusion Criteria***

- 18years of age
- Received a diagnosis of any cancer and treated with curative intent (no minimum dose) including Stage 3 & 4 gynecologic malignancies, treated in the platinum-sensitive setting
- Are within 6-12 months of completion of taxane or platinum-based chemotherapy (ie no other chemotherapeutic agents since completing of the chemotherapy regimen)
- Report > Grade 1 on the Patient Reported NCI Common Terminology Criteria for Adverse Events version 5.0 grading scale (numbness and tingling severity item) and neuropathic pain >3 on the DN4 (interview) (0-7)
- The presence of peripheral neuropathy due to chemotherapy (following onset of chemotherapy), as established via clinical assessment.
- May be on maintenance oncologic therapies (ie endocrine therapy, PARP inhibitors) not known to cause neuropathy
- No current plans for chemotherapy in the next 6 months
- Currently engaging in < 90min per week of planned moderate-intensity aerobic exercise
- Independent with ambulation and transfers with or without ambulatory assistance (EGOG 0-2)
- Able to communicate sufficiently in English to complete intervention, questionnaires, and consent
- Willing to participate in the intervention and attend in-person physical assessments
- Have access to and are able to operate videoconferencing.

***Exclusion Criteria***

- Known neurological conditions influencing cognition and preventing safe or appropriate engagement with self-management and exercise recommendations
- Individuals currently using TDM1 adjuvant hormone therapy
- Pre-existing neuropathy prior to the start of chemotherapy
- Currently enrolled in other cancer rehabilitation or exercise-based programs/interventions.

**
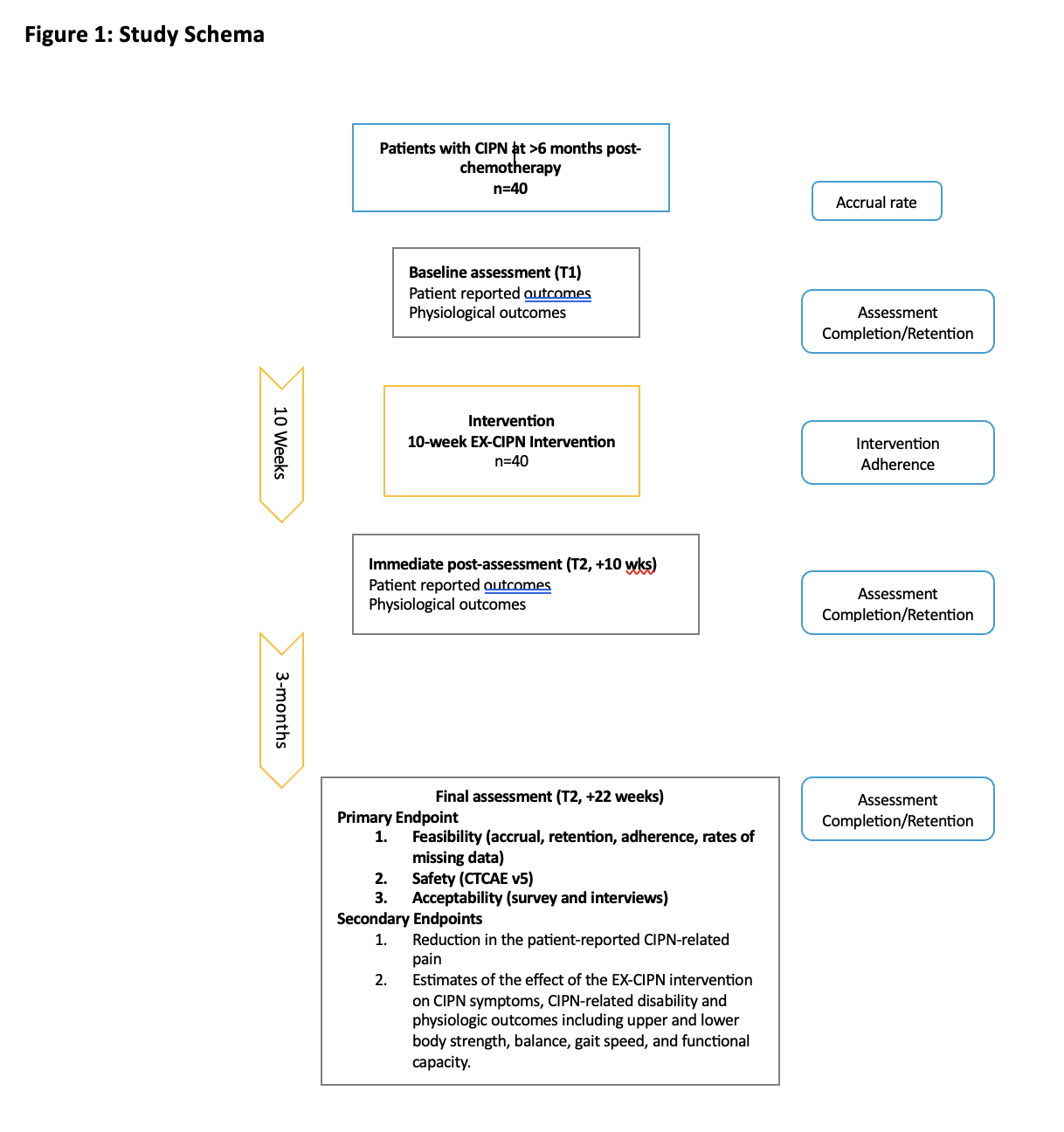
**

**STATEMENT OF COMPLIANCE**

This study will be conducted in compliance with the protocol, International Conference on Harmonisation [ICH] E6, applicable regulatory requirements, and carried out in accordance with Good Clinical Practice (GCP) as per the following:

- Tri-Council Policy Statement: Ethical Conduct for Research Involving Humans, Canadian Institutes of Health Research, Natural Sciences and Engineering Research Council of Canada, Social Sciences and Humanities Research Council of Canada
- Personal Information Protection and Electronic Documents Act [PIPEDA]

All key personnel (all individuals responsible for the design and conduct of this study) have completed GCP/Human Subjects Protection Training.

**1.0 OBJECTIVES**

**1.1 Primary Objectives**

- Primary Objective 1: *To determine if EX-CIPN is feasible, acceptable and safe in cancer survivors experiencing persistent CIPN.*
- Primary Objective 2: *To examine the feasibility of the study design and methods (recruitment and retention rates, feasibility of data collection and procedures).*

**1.2 Secondary Objective**

- Secondary Objective: *To obtain estimates of the effects of EX-CIPN on pain, CIPN symptoms, CIPN related disability and physiologic outcomes including upper and lower body strength, balance, gait speed, and functional capacity.*

**2.0 BACKGROUND and Rationale**

Improvements in early detection and treatment of cancer have led to a substantial increase in the number of people living with a personal history of cancer^10^. In 2018, there were an estimated 43.8 million cancer survivors diagnosed within the previous 5 years worldwide and this number is expected to grow by close to 25% over the next decade^11^. With increasing proportions of people now surviving cancer and transitioning into the extended survival phases of cancer care, the long-term effects of cancer and its treatments and previously unrecognized chronic morbidity and related disability are of increasing importance^12,13^.

Chemotherapy is a necessary component to increase the cure of many common cancers; however, it can induce changes to cellular structure and functions resulting in toxic effects ^14^. Chemotherapy-induced peripheral neurotoxicity (CIPN) is a prevalent adverse effect of adjuvant/curative chemotherapy agents^15-17^ which can develop at any point during chemotherapy receipt or occasionally after treatment completion^18^. CIPN is estimated to be present in 2/3 of patients who receive neurotoxic chemotherapy. Acute CIPN generally improves over the first 3-6 months following treatment completion^19,20^. However, in 30-40% of patients (higher for those treated with taxanes and platinum analogues), CIPN signs and symptoms such as neuropathic pain or decreased sensation can persist for months or years post-treatment^19,21-24^ .

While the exact pathogenesis of CIPN is unknown, CIPN is considered a predominately sensory neuropathy that typically affects distal limbs but can also be accompanied by motor neuropathy manifesting as weakness and autonomic neuropathies which may impact cardiovascular performance^25^. The sensory involvement of CIPN can result in pain, numbness, paresthesia (tingling, burning sensation), temperature sensitivity and/or proprioception^26^. These symptoms can lead to statis and dynamic instability, gait disturbances, and increased appendicular muscle weakness, resulting in physical deconditioning and an increased risk of falls. The risk of falls in cancer patients with CIPN is almost double compared to individuals without CIPN^27^. **Therefore, CIPN has a profound impact on the quality of life for individuals who have undergone treatment for cancer, including pain, weakness, physical disability and impairment, falls, compromised social well-being, and obstacles in returning to work after treatments are completed**^28-33^**.**

Currently, there are no evidence-based approaches for preventing CIPN^34^ and symptomatic treatment options for established CIPN are limited and of uncertain benefit^35-38^. Pharmacological agents that are effective for the symptomatic treatment of diabetic and HIV-related neuropathies, such as tricyclic antidepressants and antiepileptic drugs, have generally yielded negative results with CIPN^37-40^ and require more caution when used for older adults with cancer^41^ . Duloxetine is currently the only recommended pharmacological agent for the symptomatic treatment of CIPN^35^, though its benefit is limited and studies have not compared its use across all types of cancer or neuropathy causing agents^35^. Other neurological agents including gabapentin and pregabalin have also been tested to prevent or treat CIPN in clinical trials, but the results have not supported their use and their use is not recommended outside clinical trials^42^. These medications can result in side effects including dry mouth, constipation, diarrhea, and dizziness^43^. Further, their use may be contraindicated with tamoxifen, a common endocrine therapy in women with breast cancer. Given the lack of benefits and side effects of pharmacologic therapies along with cancer survivors’ desire to limit any further required medication^44^, research examining a wide range of non-pharmacologic interventions has emerged^45^. Of these, the most recent evidence suggests that physical therapy/exercise and acupuncture may reduce CIPN symptoms including pain^35,45,46^; however, larger definitive studies are needed to confirm the efficacy and clarify the risks of these interventions^35^.

Exercise-based rehabilitative interventions are effective for managing common side effects of treatment and enhancing functional abilities in individuals affected by cancer, both during and after treatment^47,48^. Recent evidence has also shown that exercise is feasible and may be an effective intervention for different forms of peripheral neuropathy^1,2^ and for patients experiencing CIPN^3-7^. Exercise may potentially alleviate peripheral neuropathy through several neurophysiological mechanisms in the peripheral and central nervous systems, including the induction of an anti-inflammatory environment, increasing the supply of blood, glucose and oxygen to mitochondria, and by affecting psychosocial processes^49-52^. Observational trials have demonstrated connections between higher levels of physical activity and milder cases of CIPN ^53,54^. Preliminary evidence on exercise-based rehabilitation symptomatic interventions for the treatment of established CIPN, while modest and heterogeneous in terms of the intervention protocols tested and outcomes measured, demonstrate encouraging positive effects on CIPN-related outcomes^3-7^. However, professional organizations such as the American Society of Clinical Oncology (ASCO) and the European Society for Medical Oncology (ESMO) have not made any strong recommendations on the use of exercise-based interventions for the prevention or symptomatic treatment of CIPN. **In its most recently updated guideline, ASCO stated that “preliminary supportive evidence” exists in favor of exercise to prevent and treat CIPN, but concluded that “no recommendation can be made” due to the lack of robust evidence and recommends that more rigorous definitive studies are needed to confirm efficacy and clarify risks^35^.**

To date, the evidence on exercise intervention for the prevention and symptomatic treatment of CIPN is limited by low study quality, lack of homogeneous patient population, and a lack of definitive rigorous trials with CIPN pain and symptoms as the primary end-point^2,4,7^. Further, the incorporation of behaviour change theory and related behaviour change techniques is recommended in the development and evaluation of rehabilitation interventions that require patient’s to adopt health-related behaviours^55^; however, a recent review of exercise interventions for the prevention and management of CIPN found that no study has incorporated a clear theoretical or conceptual framework related to behaviour change^56^.

Traditional supervised exercise-based rehabilitation interventions for people with cancer have been facility-based (e.g. hospital or university). Over the past decade an increasing number of supervised virtual exercise-based interventions^57^ have been shown to be safe, feasible, and effective^58-60^. These arose in response to well-documented patient barriers to attending facility-based programming, including the lack of locally available cancer-exercise programs, inflexible program hours, costs, time associated with transportation, and symptom burden^61-64^. Most people with cancer report a preference for exercising at home, if provided with appropriate support and guidance from a qualified oncology exercise professional^63,65^ and evidence demonstrates good compliance and positive experience with virtual platforms for exercise delivery^66^. The majority of exercise studies for the prevention or symptomatic treatment of CIPN have been delivered in supervised settings^67^. **There has been no study of a remotely delivered theory-based exercise-based rehabilitation intervention for the symptomatic treatment of persistent CIPN.**

In response, we developed an innovative remotely delivered 10-week exercise-based rehabilitation program (EX-CIPN) for cancer survivors with persistent CIPN. As a first step, we propose a Phase I study to explore the feasibility and acceptability of the program and methods, establish safety, and describe the preliminary effects of the program on clinical outcomes.

**2.1 How will current treatment options will be optimized as a result of this clinical trial?**

Chemotherapy-induced peripheral neuropathy is among the most common and potentially debilitating adverse effects of neurotoxic chemotherapy. There are currently very limited proven treatment options for CIPN. There is a rapidly increasing prevalence of people with cancer who will be treated and cured of cancer using these standard neurotoxic regimens and who will be impacted by this debilitating adverse effect. Effective symptomatic treatment strategies for CIPN are urgently needed to allow these people to have improved quality of life, function, and to be able to reintegrate fully into their social and vocational activities.

Exercise-based rehabilitation interventions are a potentially effective intervention for patients with persistent CIPN, though more rigorous theoretically-based studies are required in well-defined populations^2,4,7^. In addition, innovative approaches to intervention delivery that are sustainable and accessible should be considered^68-70^.

The EX-CIPN program has been developed based on previous evidence and guidance regarding CIPN and exercise^6,71-75^, exercise guidelines for cancer survivors^76^, and established behaviour change theory and techniques to promote uptake of behaviours^77-82^. The remote delivery of EX-CIPN helps address barriers to accessing and providing rehabilitation and provides a cost-effective model that can be widely adopted.

**2.2** **Preliminary results and/or studies explaining the significance and potential for success**

***Significance***

Improvements in early detection and the treatment of cancer have led to a significant increase in the number of people living with a personal history of cancer worldwide^10^. In 2018, approximately 43.8 million cancer survivors were diagnosed within the previous 5 years and this number is expected to grow by close to 25% over the next decade^11^. With increasing proportions of people now surviving cancer and transitioning into the extended survival phases of cancer care, the long-term effects of cancer and its treatments and previously unrecognized chronic morbidity and related disability are of increasing importance^12,13^.

Two-thirds of patients who receive neurotoxic chemotherapy will develop CIPN and in 30-40% of patients (higher for those treated with taxanes and platinum analogues), CIPN signs and symptoms such as neuropathic pain or decreased sensation can persist for months or years post-treatment^19,21-24^. CIPN has a profound impact on the quality of life for individuals with cancer, including pain, weakness, physical disability and impairment, falls, compromised social well-being, and obstacles in returning to work after treatments are completed^28-33^.

Accessible and effective symptomatic treatment strategies for CIPN are urgently needed to allow these people to have improved quality of life, function, and to be able to reintegrate fully into their social and vocational activities. This will be the first study of a remotely delivered exercise-based rehabilitation intervention for the symptomatic treatment of persistent CIPN.

This study is of scientific interest and clinical importance because: (a) the number of cancer survivors is rising; (b) CIPN is common persistent side effect that severely affects quality of life; (c) there are currently few symptomatic treatment options available for CIPN; and (d) virtual exercise-based rehabilitation is a promising and accessible intervention for CIPN.

***Potential for Success***

To date there have been a small number of studies on exercise-based rehabilitation for persistent CIPN. The current evidence regarding CIPN suggests that interventions that are 8-12 weeks in duration and include a combination or resistance, aerobic, balance and nerve guiding exercises are most promising^6,7,71-73,83^. The most recent systematic reviews and meta-analyses of exercise for the prevention and treatment of CIPN demonstrate encouraging reductions in neuropathic pain^71^ and CIPN symptom severity^6^, and improvements in balance performance^71^ and quality of life^71,73^. However, in all of these reviews, the authors conclude that there is a need for more definitive and rigorous trials with clearly defined populations and intervention components supported with behaviour change theory and techniques, and with CIPN as the primary outcome. The EX-CIPN program has been developed based on previous evidence and guidance regarding CIPN and exercise^6,71-75^, exercise guidelines for cancer survivors^76^, and established behaviour change theory and techniques to promote uptake of behaviours^77-82^. The remote delivery of EX-CIPN helps address barriers to accessing and providing rehabilitation and provides a cost-effective model that can be widely adopted.

In addition, our team has expertise in the development and delivery of remote cancer rehabilitation interventions^84-89^, in the assessment and treatment of chemotherapy induced peripheral neuropathy^75^, in rigorous safety assessments for exercise & rehabilitation interventions, exercise-based intervention for the prevention of CIPN^75,90^, and the development of exercise guidelines for cancer survivors^76^. Further, both PI’s have expertise in knowledge translation and implementation; therefore, if shown to be feasible, acceptable, and safe, they have the skills to move further to a Phase III study and increased uptake into practice.

**3.0 Experimental design and methods**

**3.1 Study Design**

The proposed study is a single centre single-group Phase I trial with patients who are experiencing persistent CIPN. The study protocol is reported according to the Standard Protocol Items: Recommendations for Interventional Trials [SPIRIT] 2013^92,93^ (see Table 1). A CONSORT^93^ participant flow diagram based on feasibility studies is shown in Figure 2. This study will be registered with clinicaltrials.gov and REB approval will be obtained through the University Health Network Research Ethics Board.

**3.2 Study population**

Potential participants will be recruited using several strategies:

1. Patients from Princess Margaret Cancer Centre who are experiencing CIPN will be recruited from clinics.
2. Study flyers will be distributed in several hospitals across the Greater Toronto Area.
3. The study will be promoted on Princess Margaret Cancer Centre social media channels.

**3.2.1 Inclusion Criteria**

- > 18years of age
- Received a diagnosis of any cancer and treated with curative intent (no minimum dose) including Stage 3 & 4 gynecologic malignancies, treated in the platinum-sensitive setting
- Are within 6-12 months of completion of chemotherapy (ie no other chemotherapeutic agents since completing of the chemotherapy regimen)
- Report > Grade 1 on the Patient Reported NCI Common Terminology Criteria for Adverse Events version 5.0 grading scale (numbness and tingling severity item)^94,95^ and neuropathic pain >3 on the DN4 (interview) (0-7)^96^
- The presence of peripheral neuropathy due to chemotherapy (following onset of chemotherapy), as established via clinical assessment
- May be on maintenance oncologic therapies (ie endocrine therapy, PARP inhibitors) not known to cause neuropathy
- No current plans for chemotherapy in the next 6 months
- Currently engaging in < 90min per week of planned moderate-intensity aerobic exercise
- Independent with ambulation and transfers with or without ambulatory assistance (EGOG 0-2)
- Able to communicate sufficiently in English to complete intervention, questionnaires, and consent
- Willing to participate in the intervention and attend in-person physical assessments
- Have access to and are able to operate videoconferencing.

Participants may be on medication to treat neuropathy symptoms at the start of the study, if the dose is unchanged over the past 6 weeks and symptoms of neuropathy persist. However, participants will be required to consent to not increase the dose of their neuropathy medication or start a new medication or treatment for neuropathy during the course of the study. Medication use will be tracked.

Similarly, for participants currently using alternative interventions such as acupuncture or creams they can continue these treatments as long as the intervention has been unchanged over the past 6 weeks and neuropathy symptoms still persist. Participants will be asked to keep their chosen therapy/therapies unchanged during the study.

**3.2.2. Exclusion Criteria**

Participants not meeting the criteria above will be excluded from the study. Additionally, participants will be excluded if they have:

- Known neurological conditions influencing cognition and preventing safe or appropriate engagement with self-management and exercise recommendations
- Pre-existing neuropathy prior to the start of chemotherapy
- Are currently enrolled in other cancer rehabilitation or exercise-based programs/interventions.
  1. **Recruitment**

Potential participants will be identified by clinic staff and asked if they are interested in learning more about the research study and consent to having their contact details shared with the study staff. In addition to active clinic recruitment, an email address or telephone number will be available on information flyers to connect participants to study staff. These flyers will be distributed in clinic waiting areas at Princess Margaret and other hospitals in the GTA as well as on social media.

**3.4 Screening, Enrollment, and Data Collection**

All potential participants will be provided with information on the trial. Those who are interested will undergo a screening process to determine eligibility. Individuals who meet the study criteria and provide written consent to the study will be enrolled and assigned a study identification number and scheduled to complete an in-person baseline assessment (T1). In addition, they will receive an electronic link via Research Electronic Data Capture tools (REDCap) to the baseline set of patient reported questionnaires that will be completed prior to their initial assessment and randomization (T1). Follow-up assessments will be conducted at 10 weeks (T2) (immediate post-intervention) and 22 weeks (T3) (3 month post intervention). Survey and qualitative data will be collected at the end of the program (between T2 and T3) (see Figure 1 for study schema)

During the intervention data will be collected on adherence to the program. Data will be collected from the Fitbit and Physitrack apps showing number of workouts and amount of log-ins respectively. Health coaching call attendance and self-report completion pf the exercise plan will also be collected during weekly calls in the intervention.

To optimize retention and prevent attrition, we will ensure frequent contact and monitoring with participants^3,97^, and will use a collaborative- patient-centric approach including working individually with the patient, communication of the rationale for each phase of the study, allowing flexibility in data collection methods, and promoting shared accountability and responsibility in addressing the patients’ needs.

- 1. **Withdrawal and replacement of individual participants**

Participants will be informed that they can withdraw from the study at any time without any consequences. Participants who withdraw from the study prior to the start of the intervention will be replaced. However, participants who withdraw after intervention start will not be replaced and will be asked to share the reason for withdrawing from the study.

**4.0 Study Intervention AND Procedure**

**EX-CIPN**

All participants will receive the EX-CIPN intervention. EX-CIPN has been developed based on previous evidence and guidance regarding CIPN and exercise^6,71-75,83^, exercise guidelines for cancer survivors, and established behaviour change theory and techniques to promote uptake of behaviours^77-82^. The remote delivery of EX-CIPN helps to barriers to accessing and providing rehabilitation and provides a cost-effective model that can be widely adopted. EX-CIPN is comprised of: a progressive 10-week exercise program (aerobic, resistance and balance) supported with a mobile application (Physitrack^®^) and wearable technology (Fitbit™) to track activity (see Table 2 for specific exercise components); and (2) weekly brief video/telephone check-ins provided by an oncology exercise specialist who is trained in motivational interviewing. Informed by behavior change theory, the program components aim to provide patients with the knowledge and tools needed to reach and maintain their wellness and exercise goals (see Table 3  for list of embedded behavior change techniques and tools^99^). Multiple theoretical models are integrated within the intervention (i.e., motivational interviewing, cognitive behavioral therapy, transtheoretical model of behaviour change), with the focus on addressing and resolving motivational ambivalence and identification and modification of the cognitive distortions that prevent adoption of appropriate health behaviors and addresses relapse and long-term maintenance of behavior change^77-82^. The program is provided free-of-charge to participants, other than out of pocket travel costs (i.e., public transport, parking) for in-hospital assessments.

1. Individualized progressive exercise program (See Table 2): Each participant will receive an individualized progressive exercise program that includes cardiovascular and strength training as well as balance exercises and will be adjusted to the needs of the patient. The program will aim to reach at least 150 minutes of moderate intensity aerobic exercise per week, resistance exercises 2–3 times per week focusing on major muscle groups, and flexibility stretching for major muscle groups 2–3 times per week, and desensitization exercises. The exercise program will be revised and progressed during the weekly scheduled check-ins. The exercise program will be supported by Physitrack^®^, which is an online application that allows customizable exercise prescriptions, videos of exercises, and tracking of exercise completion. The participants will be provided with an orientation to the Physitrack^®^ application. If the participant is unable to download or use Physitrack^®^, they will be provided with a detailed print out of their exercise program.
2. Remote monitoring: Fitbit^™^ devices will be used to monitor patients’ physical activity over the duration of the program. The assigned oncology exercise professional will have access to Fitbit^™^ data in order to monitor these metrics. Fitness tracker can promote behaviour change and allows for self-monitoring and feedback to the participant^100^ . Participants will be asked to wear the Fitbit^™^ for the duration of the study. The study staff will provide support to the participant as needed to set-up the device and access the Fitbit^™^ application.
3. Remote person-to-person clinical support: Participants will have scheduled remote check-ins and health coaching sessions with their assigned RKin. The addition of a person-to-person component is an important component in the delivery of online behaviour change interventions^101^ as it creates accountability, and provides an opportunity for tailored feedback and social support^102^. Check-ins will be offered via MS Teams video or telephone and scheduled on weeks 2, 3, 4, 6, and 8. During these calls, the RKin will review, adapt, and progress the exercise program as needed, guide the participant to reflect upon what has happened since their last check-in, discuss and develop goals for the following week, and identify potential barriers and solutions in achieving their goals. The RKin delivering the intervention are trained in motivational interviewing (MI) by a certified Motivational Interviewing Network Trainer and will incorporate the assessment and promotion of intrinsic motivation, development of self-efficacy, and will use a collaborative problem-solving approach^103^. MI elicits and strengthens motivation for change by identifying and addressing ambivalence and it has been shown to be effective in increasing physical activity in populations with chronic conditions including cancer^84,103,104^.

**5.0 Study Outcomes/Endpoints (see Table 4)**

**5.1 Primary Outcomes**

Feasibility of the intervention and methods include the following: 1) Recruitment and eligibility rates; 2) Retention and attrition; 3) Intervention adherence. An adapted Consolidated Standards of Reporting Trials diagram (see Figure 2) for pilot feasibility studies will be utilized to track participant flow throughout the study including initial eligibility, recording of consent, collection of baseline measures, randomization, and completion of follow-up assessments^93^. *See Section 9.0 for* *Criteria for positive trial/interpretation of results*.

To evaluate acceptability and steer future program refinement, we will ask all participants to complete a post-study survey to provide feedback on their experience. We will also conduct in-depth semi-structured qualitative video interviews (MS Teams) with a sub-sample of approximately 1/3 of participants following the T2 assessment. The purpose of the qualitative interviews is to gain feedback on program components, how participants engaged with the program, and in what ways the program impacted their lives^105^. All interviews will be conducted using a semi-structured interview guide to address feasibility and acceptability issues specific to EX-CIPN. All interviews will be digitally recorded and transcribed verbatim. An interpretive descriptive qualitative methodology will be used^105-109^.

- Accrual rate will be assessed based on CONSORT criteria^93^ through a screening log that tracks data collected of all screened patients. Eligibility screening will be performed to identify eligible consented and eligible non-recruited individuals with non-recruitment reasons documented. Number of participants consented and enrolled will be tracked each month.
- Retention rates at each study time point will be monitored. Retention rates will be calculated as the proportion of participants that attend each assessment time point (T1, T2, and T3). We will also examine rates of complete and missing data.
- Adherence to the intervention will be assessed through health coaching call attendance, Fitbit™ usage, and self-report completion of weekly exercise plan (determined during weekly calls).
- Safety will be assessed throughout the study. All adverse events will be scored on the CTCAE version 5.0^9^ and documented during weekly appointments and at follow-up assessments with the RKin. Assessment tests and all exercises will be stopped at any time if any pain or discomfort is experienced.

**5.2 Secondary Outcomes**

Patient-reported outcomes measures and physiologic assessments will be completed at baseline (T1), at the end of the intervention, 10 weeks (T2), and 3 months post-intervention, 22 weeks (T3) (see Figure 2). Patient-reported outcomes will be completed online via RedCap™ or on paper at each time point. The specific measures include the numeric pain rating scale (pain)^110,111^, the EORTC CIPN-20 (CIPN symptoms)^112^, CIPN-RODS (CIPN-related disability)^113^. Physiologic outcomes will include upper (grip dynamometer) and lower body strength (sit-to-stand test), balance (Berg Balance Test), gait speed, and functional capacity (6-minute walk test).

**6.0 Sample Size and timeline**

While there is no overall consensus regarding appropriate sample size in a feasibility study^115^, based on a simulation of a range of sample sizes and values of standard deviation for precision of estimate (α=0.05 and power at 80%); 35-40 is at the elbow point of the curves (see Figure 3). Therefore, a sample size of 40 participants will be included in the study, which is considered large enough to examine the feasibility of the study^116,117^.

There are approx. 400-500 cancer patients who receive either platinum or taxane-based curative/(neo)-adjuvant chemotherapy per year at the Princess Margaret Cancer Centre per year. It is estimated that 30-40% of these patients will report CIPN > 6 months post-treatment^21^. Based on this, we anticipate recruiting 5-6 participants per month (See Figure 4). The study will take 15 months for accrual and 20 months for data collection.

**7.0 Data management**

A detailed database will be developed to track each participant’s progress and will include notes on any clinical or study deviations. All study data will be stored in encrypted files on secure servers at the University Health Network and outcome data will be stored on REDCap® which is a secure web-based application for electronic data. Data files will be restricted to study investigators and authorised study personnel. Data quality and integrity will be assured using data audits, access control, and monitoring and cleaning data.

**8.0 Analyses**

**8.1 Primary outcome**

Study feasibility will be evaluated using descriptive statistics and will include: 1) the rate of recruitment (average number of patients per month) 2) the number and proportion of patients who are eligible (and reasons for ineligibility); 3) the number who provide consent (and reasons for declining); 4) attrition rates at each time point (and reasons for drop out); and, 5) capture of outcome data at each time point and missing items. The feasibility of the intervention will be assessed by examining: 1) Fitbit^TM^ and Physitrack usage; and 2) Weekly virtual check-in attendance.

Acceptability will be assessed qualitatively through the interview data and a thematic analysis will be conducted^119^. Analyses will be primarily deductive with a number of categories pre-selected to align with the main program components as well as program strengths, weaknesses, and areas of improvement. Once this initial coding is complete, the interviews will be read once again, and any additional codes will be derived inductively. Themes will be generated by a close examination of codes and categories, and the relationships between them, and discussions with the research team.

Any safety event related to the program will be reported using Common Terminology Criteria for Adverse Events v5.0^9^ .

**8.2 Secondary Outcomes**

Capture rates of the patient-reported outcomes will be assessed and described at each time point overall and for each treatment arm to inform future sample size calculations. The distribution of baseline values (mean/standard deviation and median/interquartile range) will be summarised for all outcomes. To inform sample size calculations for a phase I trial Within-person change from baseline to follow-up (T3-T1) will be calculated and summarised for all outcomes. To understand how Ex-CIPN affects change, mixed effects models will be used to estimate the change from baseline to post-intervention and from post-intervention to follow-up using all available data for each patient (allowing us to incorporate data from patients who withdraw from the study). Separate models will be run for each of the secondary outcomes to provide information about the magnitude and timing of change for each outcome. Data will be analyzed when all recruitment and data collection has been completed.

**9.0 CRITERIA FOR POSITIVE TRIAL/Interpretation of Results**

We will define our intervention as feasible to test in a larger, multi-center RCT if there is reasonable recruitment of 4-5 participants per month^7,122^, retention of 70% at each time point ^7,72^ and reasonable adherence (70%) to the intervention components (including Fitbit usage, call attendance, and completion of weekly exercise plans)^7^. In addition, success will be measured by high levels of treatment acceptability based on survey and qualitative interview data. Safety of the intervention will be confirmed if no serious adverse events (defined as anything above a Grade 2 of the CTCAE v5) related to participation in the intervention occur. If any one of those criteria is not met, we will make appropriate modifications to the protocol prior to a larger RCT. Calculation of the sample size for a larger RCT will be based on a minimally important clinical difference of 2 points between the T1 and T3 on the numeric pain rating scale^111^ .

# **10.0 Data Collection, Handling, and Storage**

**10.1 Data Collection**

Study data will be collected from patient-reported outcomes/ questionnaires and/or participant’s medical records, and transcribed on case report forms (CRFs). These forms can be paper, electronic, or both. The medical records will be reviewed at study-specific timelines/visits for the collection of results from routine clinical tests that are carried out as part of standard of care.

Study data will be under secure password or kept under lock and key in the research office.

**10.2 Data Handling**

Patient registration and outcomes reporting will be through a de-identified database web-based accessible for registered investigators through research database (REDCap). The research database provides an environment for collecting, managing and reporting outcome data and enables the flexibility to meet the unique requirements of this study. The application also provides an extensive administrative interface, with capabilities to create, design and manage clinical research forms. Furthermore, it is fully compliant with privacy regulations (HIPAA/PIPEDA), with enhanced privacy-related features including audit logging capabilities, role-based user access permissions, and a repository for study-related source documentation. Any source documents that contain Personal Health Information (PHI) will be properly de-identified prior to being uploaded to the research database.

Hard copy study files will be kept secured. All computerized files will be password protected. Coded study data will be stored on research servers.

**10.3 Storage and Record Retention**

Study data will be stored and archived as follows:

1. Hard copies: consent forms, questionnaires, coordinator source documentation
2. Electronic data: medical records (EPR/EPIC), images (Coral viewer, PACS), all other data (RMP network), regulatory files

In accordance with institutional policies, all records and documents pertaining to the study will be retained by the study trial site for 10 years from the completion of the study, and will be available for inspection by institutional representatives (e.g. REB, CCRU Quality Assurance), sponsor, Health Canada, and other regulatory bodies. No clinical trial records will be destroyed without the documented consent of the PI.

**10.4 Data Safety and Monitoring Plan**

The PI will assume primary responsibility for monitoring the progress of the trial and the safety of participants.

As part of UHN-PM, this trial may be selected at random to be monitored by the staff of the data safety and monitoring board. Data will also be submitted to the UHN REB annually for continuing review and at the completion of the study.

# **11.0 Ethics**

**11.1 Research Ethics Board (REB)**

The study will be submitted for initial and ongoing annual REB review at the University health Network (Panel C). Study related procedures will not commence prior to receipt of REB approval. All study protocols, consent forms, and patient-facing material will receive REB approval before being used. Any amendment to the study will be submitted for review by the REB before any changes are implemented unless required to eliminate immediate hazard to the study participants.

**11.2 Informed Consent Process**

If a potential participant has expressed interest in the study, a qualified study team member will discuss the study details and consent form with the potential participant either in-person or remotely (e.g. telephone, MS Teams). This discussion will involve careful explanation of all items outlined in the consent form, the investigational nature of the study, any benefits and risks to research participants. Sufficient time will be given to the potential participant to answer any questions.

Written informed consent will be obtained prior to the participant beginning any study-related procedures.

If new information becomes available during the study, study participants will be informed of the changes and asked to re-consent by study staff, if required.

**11.2.1 REDCap eConsent**

If in-person consenting is unavailable, then remote consenting can be completed using the UHN Approved REDCap eConsent Process. Potential participants will be contacted via telephone to conduct the informed consent discussion. A standard script will be used to ensure the same process is followed for every participant. After all the participant’s questions have been answered, they will be sent a REDCap link via email. A member of the research team will assist the participant when completing the eConsent form.

**11.3 Protocol Deviations**

A protocol deviation is any noncompliance with the clinical trial protocol and/or Good Clinical Practice (GCP). The noncompliance may be either on the part of the subject, the investigator, or the study site staff. The study PI and study staff are responsible for knowing and adhering to the study protocol and the reporting requirements of relevant regulatory agencies (e.g. UHN REB). In the event a deviation occurs, the details and reason(s) for the deviation, as well as any effect on patients’ safety and/or trial data, must be documented. All deviations from the protocol will be addressed in study subject source documents. Corrective and preventative actions will be developed by the site and implemented promptly. If the deviation meets the reporting requirements of the relevant regulatory agencies, the deviation will be reported in a timely manner. These practices are consistent with ICH E6.

#

# **12.0 Direct Access to Source Data/Documents**

- Only the investigators and the study team will have access to study-related source data/documents.
- The link of study number and identifiable information (e.g. Medical Records Number) will be stored separately from the remainder of the study information and not be transported or transmitted outside the research office.

# **13.0 Publication and data avaiLability Policy**

Data derived from this study may be reported in scientific publications. Patients will not be indicated by name in any publications or presentation that may result from this study. Study data will be available upon request (following REB approval).

# **14.0 Time to Study Initiation and Milestone Achievements**

| **Objectives** | **Milestones** | **Start Date** | **Completion Date** | **Describe how you are going to monitor progress (Deliverables)** |
| --- | --- | --- | --- | --- |
| Develop research protocol | Completion of study design and protocol | April, 2023 | Sept, 2023 | Completion of one clinical trial research protocol |
| Obtain Research Ethics Board (REB) approval | REB submission and approval | Oct 2023 | Jan 2024 | Receipt of approval by REB at PM |
| Register the clinical trial and publish study protocol | Registration of the clinical trial on ClinicalTrials.gov  Manuscript completion, submission, acceptance. | Nov 2023 | Feb 2024 | ClinicalTrials.gov posting  Protocol article published |
| Train research staff | Training Rkin to deliver intervention and MSc student to run study | Nov 2023 | Feb 2024 | Good Clinical Practice (GCP) and relevant technical training certificates of research staff |
| Initiate the trial | Activation of trial | Feb 2024 | Feb 2024 | First patient enrolment |
| Monitor trial accrual | Completion of trial accrual | Dec 2023 | Mar 2025 | Completion of 50% of total target accrual by Aug, 2024.  Completion of 100% of total target accrual by Mar, 2025. |
| Complete follow-up data collection | Data collection completion | Apr 2024 | Aug 2025 | Completion of 50% of follow up data collection by Jan, 2025.  Completion of 100% of follow up data collection by Aug, 2025. |
| To report/ disseminate trial results in a timely manner | Abstract submission and presentation | Sept 2025 | Dec 2025 | Conference abstract publications submitted for 1 national, and 1 international meeting with patients, healthcare providers, and/or relevant stakeholders in the audience. |
| To publish trial results | Manuscript completion, submission, and acceptance. Reporting of results on ClinicalTrials.gov. | Sept 2025 | Dec 2025 | One article published ClinicalTrials.gov results posting. |

**References**

1. Dixit S, Maiya A, Shastry B. Effect of aerobic exercise on peripheral nerve functions of population with diabetic peripheral neuropathy in type 2 diabetes: a single blind, parallel group randomized controlled trial. *J Diabetes Complications.* 2014;28(3):332-339.

2. Streckmann F, Zopf E, Lehmann H, et al. Exercise intervention studies in patients with peripheral neuropathy: a systematic review. . *Sport Med.* 2014;44(9):1289-1304.

3. Perlis RH. Abandoning personalization to get to precision in the pharmacotherapy of depression. *World Psychiatry.* 2016;15(3):228-235.

4. Brayall P, Donlon E, Doyle L, Leiby R, Violette K. Physical therapy-based interventions improve balance, function, symptoms, and quality of life in patients with chemotherapy-induced peripheral neuropathy: a systematic review. *Rehabil Oncol.* 2018;36(3):161-166.

5. Duregon F, Vendramin B, Bullo V, et al. Effects of exercise on cancer patients suffering chemotherapy-induced peripheral neuropathy undergoing treatment: a systematic review. *Critical reviews in oncology/hematology.* 2018;121:90-100.

6. Lin W, Wang R, Chou F, Feng I, Fang C, Wang H. The effects of exercise on chemotherapy-induced peripheral neuropathy symptoms in cancer patients: a systematic review and meta-analysis. *Support Care Cancer.* 2021;29:5303-5311.

7. Kanzawa-Lee G, Larson J, Resnicow K, Smith E. Exercise Effects on Chemotherapy-Induced Peripheral Neuropathy: A Comprehensive Integrative Review. *Cancer Nursing* 2020;43(3):E172-E185.

8. Schulz K, Altman D, Moher DftCG. CONSORT 2010 Statement: updated guidelines for reporting parallel group randomised trials. . *Ann Int Med.* 2010;152.

9. Colevas A, A. S. The NCI Common Terminology Criteria for Adverse Events (CTCAE) v 3.0 is the new standard for oncology clinical trials. . *Journal of Clinical Oncology* 2004;22(14).

10. Allemani C, Weir H, Carreira H, et al. Global surveillance of cancer survival 1995–2009: analysis of individual data for 25 676 887 patients from 279 population-based registries in 67 countries (CONCORD-2). *The Lancet.* 2015;385(9972):977-1010.

11. Ferlay J, Ervik M, Lam F, et al. Global Cancer Observatory: Cancer Today. 2020; <https://gco.iarc.fr/today>,, 2023.

12. Collaboration GBoDC. Global, Regional, and National Cancer Incidence, Mortality, Years of Life Lost, Years Lived With Disability, and Disability-Adjusted Life-Years for 29 Cancer Groups, 1990 to 2016: A Systematic Analysis for the Global Burden of Disease Study. *JAMA Oncology.* 2018;4(11):1553-1568.

13. Nekhlyudov L, Campbell G, Schmitz K, et al. Cancer-related impairments and functional limitations among long-term cancer survivors: Gaps and opportunities for clinical practice. . *Cancer.* 2022;128(2):222-229.

14. Amjad M, Chidharla A, Kasi A. *Cancer Chemotherapy. .* Treasure Island (FL): StatPearls Publishing LLC; 2023.

15. Carozzi V, Canta A, Chiorazzi A. Chemotherapy-induced peripheral neuropathy: What do we know about mechanisms? . *Neuroscience letters.* 2015;596:90-107.

16. Kanzawa-Lee G. Chemotherapy-induced peripheral europathy: nursing implications. *J Infus Nurs* 2020;43:155–166.

17. Hausheer F, Schilsky R, Bain S, Berghorn E, Lieberman F. Diagnosis, management, and evaluation of chemotherapy-induced peripheral neuropathy. *Semin Oncol.* 2006;33(1):15-49.

18. Molassiotis A, Cheng H, Leung K, et al. Risk factors for chemotherapy-induced peripheral neuropathy in patients receiving taxane-and platinum-based chemotherapy. *Brain Behav* 2019;9(6).

19. Seretny M, Currie GL, Sena ES, et al. Incidence, prevalence, and predictors of chemotherapy-induced peripheral neuropathy: A systematic review and meta-analysis. *Pain.* 2014;155(12):2461-2470.

20. Simon N, Danso M, Alberico T, Basch E, Bennett A. The prevalence and pattern of chemotherapy-induced peripheral neuropathy among women with breast cancer receiving care in a large community oncology practice. *Quality of life research : an international journal of quality of life aspects of treatment, care and rehabilitation.* 2017;26(10):2763–2772.

21. Molassiotis A, Cheng H, Lopez V, et al. Are we mis-estimating chemotherapy-induced peripheral neuropathy? Analysis of assessment methodologies from a prospective, multinational, longitudinal cohort study of patients receiving neurotoxic chemotherapy. . *BMC cancer.* 2019;19(1).

22. Hou S, Huh B, Kim H, Kim K, Abdi S. Treatment of chemotherapy-induced peripheral neuropathy: systematic review and recommendations. . *Pain Physician.* 2018;21(6):571-592.

23. Pereira S, Fontes F, Sonin T, et al. Chemotherapy-induced peripheral neuropathy after neoadjuvant or adjuvant treatment of breast cancer: a prospective cohort study. . *Support Care Cancer* 2016;24(4):1571-1581.

24. Eckhoff L, Knoop A, Jensen M, Ewertz M. Persistence of docetaxel-induced neuropathy and impact on quality of life among breast cancer survivors. . *Eur J Cancer.* 2015;51(3):292-300.

25. von Hehn C, Baron R, Woolf C. Deconstructing the neuropathic pain phenotype to reveal neural mechanisms. *Neuron.* 2012;73(4):638-652.

26. Brozou V, Vadalouca A, Zis P. Pain in platin-induced neuropathies: a systematic review and meta-analysis. . *Pain Ther.* 2018;7(1):105-119.

27. Winters-Stone K, Horak F, Jacobs P, et al. Falls, functioning, and disability among women with persistent symptoms of chemotherapy-induced peripheral neuropathy. *J Clin Oncol.* 2017;35(23):2604-2612.

28. Shimozuma K, Ohashi Y, Takeuchi A, et al. Taxane-induced peripheral neuropathy and health-related quality of life in postoperative breast cancer patients undergoing adjuvant chemotherapy: N-SAS BC 02, a randomized clinical trial. . *Supportive Care in Cancer.* 2012;20:3355-3364.

29. Girach A, Julian T, Varrassi G, Paladini A, Vadalouka A, P. Z. Quality of life in painful peripheral neuropathies: a systematic review. *Pain Res Manag.* 2019;2019.

30. Mols F, Beijers T, Lemmens V, van den Hurk C, Vreugdenhil G, van de Poll-Franse L. Chemotherapy-induced neuropathy and its association with quality of life among 2- to 11-year colorectal cancer survivors: results from the population-based PROFILES registry. *J Clin Oncol* 2013;31:2699-2707.

31. van de Graaf D, Engelen V, de Boer A, et al. Experiences of cancer survivors with chemotherapy-induced peripheral neuropathy in the Netherlands: symptoms, daily limitations, involvement of healthcare professionals, and social support. . *J Cancer Surviv.* 2023.

32. Miaskowski C, Mastick J, Paul S, et al. Chemotherapy-induced neuropathy in Cancer survivors. . *J Pain Symptom Manag* 2017;54(2):204-218.

33. Gewandter J, Fan L, Magnuson A, et al. Falls and functional impairments in cancer survivors with chemotherapy-induced peripheral neuropathy (CIPN): a University of Rochester CCOP study. . *Support Care Cancer.* 2013;21(7):2059-2066.

34. Albers J, Chaudhry V, Cavaletti G, Donehower R. Interventions for preventing neuropathy caused by cisplatin and related compounds. . *Cochrane Database of Systematic Reviews.* 2007;24(1).

35. Loprinzi C, Lacchetti C, Bleeker J, et al. Prevention and Management of Chemotherapy-Induced Peripheral Neuropathy in Survivors of Adult Cancers: ASCO Guideline Update. . *J Clin Oncol.* 2020;38(28):3325-3348.

36. Staff N, Grisold A, Grisold W, Windebank A. Chemotherapy-induced peripheral neuropathy: a current review. *Annals of neurology.* 2017;81(6):772-781.

37. Liampas A, Rekatsina M, Vadalouca A, Paladini A, Varrassi G, Zis P. Pharmacological management of painful peripheral neuropathies: a systematic review. *Pain Ther.* 2021;10(1):55-68.

38. Majithia N, Temkin S, Ruddy K, Beutler A, Hershman D, Loprinzi C. National Cancer Institute-supported chemotherapy-induced peripheral neuropathy trials: outcomes and lessons. . *Support Care Cancer.* 2016;24(3):1439-1447.

39. Rao R, Michalak J, Sloan J, et al. Efficacy of gabapentin in the management of chemotherapy‐induced peripheral neuropathy: a phase 3 randomized, double‐blind, placebo‐controlled, crossover trial (N00C3). *Cancer.* 2007;110(9):2110-2118.

40. Rao R, Flynn P, Sloan J, et al. Efficacy of lamotrigine in the management of chemotherapy‐induced peripheral neuropathy: A phase 3 randomized, double‐blind, placebo‐controlled trial, N01C3. . *Cancer: Interdisciplinary International Journal of the American Cancer Society.* 2008;112(12):2802-2808.

41. Wasilewski A, Mohile N. Meet the expert: How I treat chemotherapy-induced peripheral neuropathy. . *Journal of Geriatric Oncology.* 2021;12(1):1-5.

42. Li Y, Lustberg M, Hu S. Emerging Pharmacological and Non-Pharmacological Therapeutics for Prevention and Treatment of Chemotherapy-Induced Peripheral Neuropathy. *Cancers.* 2021;13(4).

43. Tham A, Jonsson U, Andersson G, Soderlund A, Allard P, Bertilsson G. Efficacy and tolerability of antidepressants in people aged 65 years or older with major depressive disorder—a systematic review and a meta-analysis. . *J Affect Disord.* 2016;205:1-12.

44. Arch J, Vanderkruik R, Kirk A, Carr A. A closer lens: Cancer survivors' supportive intervention preferences and interventions received. Psychooncology. . *Psychooncology.* 2018;27(5):1434-1441.

45. Papadopoulou M, Stamou M, Bakalidou D, et al. Non-pharmacological Interventions on Pain and Quality of Life in Chemotherapy Induced Polyneuropathy: Systematic Review and Meta-Analysis. *In Vivo.* 2023;37(1):47-56.

46. D’Souza R, Alvarez G, Dombovy-Johnson M, Eller J, Abd-Elsayed A. Evidence-Based Treatment of Pain in Chemotherapy-Induced Peripheral Neuropathy. *Curr Pain Headache Rep.* 2023;27(5):99-116.

47. Mustian K, Sprod L, Palesh O, et al. Exercise for the management of side effects and quality of life among cancer survivors. . *Curr Sports Med Rep* 2009;8(6):325-330.

48. van Waart H, Stuiver M, van Harten W, et al. Effect of low-intensity physical activity and moderate- to high-intensity physical exercise during adjuvant chemotherapy on physical fitness, fatigue, and chemotherapy completion rates: results of the PACES randomized clinical trial. . *J Clin Oncol.* 2015;33(17):1918-1927.

49. Cooper M, Kluding P, Wright D. Emerging Relationships between Exercise, Sensory Nerves, and Neuropathic Pain. . *Front Neurosci.* 2016;10.

50. Chung K, Park S, Streckmann F, et al. Mechanisms, Mediators, and Moderators of the Effects of Exercise on Chemotherapy-Induced Peripheral Neuropathy. *Cancers.* 2022;14(5).

51. Gleeson M, Bishop N, Stensel D, Lindley M, Mastana S, Nimmo M. The anti-inflammatory effects of exercise: mechanisms and implications for the prevention and treatment of disease. . *Nat Rev Immunol* 2011;11:607-615.

52. Tofthagen C, Overcash J, K. K. Falls in persons with chemotherapy-induced peripheral neuropathy. . *Support Care Cancer* 2012;20:583-589.

53. Mols F, Beijers A, Vreugdenhil G, Verhulst A, Schep G, Husson O. Chemotherapy-induced peripheral neuropathy, physical activity and health-related quality of life among colorectal cancer survivors from the PROFILES registry. *J Cancer Surviv.* 2015;9(3):512-522.

54. Greenlee H, Hershman D, Shi Z, et al. BMI, lifestyle factors and taxane-induced neuropathy in breast cancer patients: the pathways study. . *J Natl Cancer Inst.* 2016;109 (2).

55. Hart T, Dijkers M, Whyte J, et al. A theory-driven system for the specification of rehabilitation treatments. . *Arch Phys Med Rehab.* 2019;100(1):172-180.

56. Tanay M, Armes J, Moss-Morris R, AM R, Robert G. A systematic review of behavioural and exercise interventions for the prevention and management of chemotherapy-induced peripheral neuropathy symptoms. *J Cancer Surviv* 2023;17:254-277.

57. Correia I, Cardoso V, Cargaleiro C, et al. Effects of home-based exercise programs on physical fitness in cancer patients undergoing active treatment: a systematic review and meta-analysis of randomized controlled trials. *Journal of Science and Medicine in Sport.* 2023.

58. Batalik L, Winnige P, Dosbaba F, Vlazna D, Janikova A. Home-Based Aerobic and Resistance Exercise Interventions in Cancer Patients and Survivors: A Systematic Review. *Cancers (Basel).* 2021;13(8).

59. Huizinga F, Westerink N, Berendsen A, et al. Home-based Physical Activity to Alleviate Fatigue in Cancer Survivors: A Systematic Review and Meta-analysis. . *Med Sci Sports Exerc.* 2021;53(12):2661-2674.

60. Kraemer M, Priolli D, Reis I, Pelosi A, Garbuio A, Messias L. Home-based, supervised, and mixed exercise intervention on functional capacity and quality of life of colorectal cancer patients: a meta-analysis. *Sci Rep.* 2022;12(1).

61. Blaney JM, Lowe-Strong A, Rankin-Watt J, Campbell A, Gracey JH. Cancer survivors' exercise barriers, facilitators and preferences in the context of fatigue, quality of life and physical activity participation: a questionnaire-survey. *Psycho-Oncology.* 2013;22(1):186-194.

62. Ottenbacher A, Day R, Taylor W, et al. Exercise among breast and prostate cancer survivors-what are their barriers? *J Cancer Surviv.* 2011;5(4):413-419.

63. Wong J, McAuley E, Trinh L. Physical activity programming and counseling preferences among cancer survivors: a systematic review. . *International Journal of Behavioral Nutrition and Physical Activity.* 2018;15(1).

64. Ormel H, van der Schoot G, Sluiter W, Jalving M, Gietema J, Walenkamp A. Predictors of adherence to exercise interventions during and after cancer treatment: a systematic review. . *Psychooncology.* 2018;7(3):713-724.

65. Gonzalo-Encabo P, Wilson R, Kang D, Normann A, Dieli-Conwright C. Exercise oncology during and beyond the COVID-19 pandemic: Are virtually supervised exercise interventions a sustainable alternative? . *Crit Rev Oncol Hematol* 2022;174.

66. Morrison K, Paterson C, Toohey K. The feasibility of exercise interventions delivered via telehealth for people affected by cancer: a rapid review of the literature. . *InSeminars in oncology nursing.* 2020;36(6).

67. Bruera E, Miller L, McCallion J, Macmillan K, Krefting L, Hanson J. Cognitive failure in patients with terminal cancer: a prospective study. *Journal of Pain & Symptom Management.* 1992;7(4):192-195.

68. Strecher V. Internet methods for delivering behavioral and health-related interventions (eHealth). *Annu Rev Clin Psychol.* 2007;27(3):53-76.

69. Alfano C, Leach C, Smith T, et al. Equitably improving outcomes for cancer survivors and supporting caregivers: a blueprint for care delivery, research, education, and policy. *CA Cancer J Clin.* 2019;69(1):35-49.

70. Alfano C, Kent E, Padgett L, Grimes M, de Moor J. Making Cancer Rehabilitation Services Work for Cancer Patients: Recommendations for Research and Practice to Improve Employment Outcomes. *PM R* 2017;9(9S2):S398-S406.

71. Guo S, Han W, Wang P, Wang X, Fang X. Effects of exercise on chemotherapy-induced peripheral neuropathy in cancer patients: a systematic review and meta-analysis. *J Cancer Surviv.* 2023;17(2):318–331.

72. Nuñez de Arenas-Arroyo S, Cavero-Redondo I, Torres-Costoso A, Reina-Gutiérrez S, Lorenzo-García P, Martínez-Vizcaíno V. Effects of exercise interventions to reduce chemotherapy-induced peripheral neuropathy severity: A meta-analysis. . *Scand J Med Sci Sports.* 2023;33(7):1040-1053.

73. Lopez-Garzon M, Cantarero-Villanueva I, Postigo-Martin P, González-Santos A, Lozano-Lozano M, Galiano-Castillo N. Can Physical Exercise Prevent Chemotherapy-Induced Peripheral Neuropathy in Patients With Cancer? A Systematic Review and Meta-analysis. *Archives of Physical Medicine and Rehabilitation.* 2022;103(11):2197-2208.

74. Kleckner I, Park S, Streckmann F, Wiskemann J, Hardy S, Mohile N. Clinical and Practical Recommendations in the Use of Exercise, Physical Therapy, and Occupational Therapy for Chemotherapy-Induced Peripheral Neuropathy. In: Lustberg M, Loprinzi C, eds. *Diagnosis, Management and Emerging Strategies for Chemotherapy-Induced Neuropathy* Springer; 2021:243-252.

75. Stoller S, Capozza S, Alberti P, Lustberg M, Kleckner I. Framework to leverage physical therapists for the assessment and treatment of chemotherapy-induced peripheral neurotoxicity (CIPN). *Support Care Cancer.* 2023;31(5).

76. Campbell K, Winters-Stone K, Wiskemann J, et al. Exercise Guidelines for Cancer Survivors: Consensus Statement from International Multidisciplinary Roundtable. . *Med Sci Sports Exerc.* 2019;51(11):2375-2390.

77. Rollnick S, Miller W, Butler C. *Motivational Interviewing in Health Care: Helping Patients Change Behavior.* New York, NY: Guilford Press; 2007.

78. Brewin C. Theoretical foundations of cognitive-behavioral therapy for anxiety and depression. . *Annu Rev Psychol.* 1996;47:33-57.

79. Ajzen I. The theory of planned behavior. *Organizational Behavior and Human Decision Processes.* 1991;50(2):179-211.

80. Bandura A. Self-efficacy: Toward a unifying theory of behavioral change. *Psychol Rev.* 1977;84(2):191-215.

81. Marlatt G, Gordon J. *Relapse Prevention: Maintenance Strategies in the Treatment of Addictive Behaviors.* New York, NY: Guilford Press; 1985.

82. Parks G, Anderson B, Marlatt G. *Relapse Prevention Therapy in the Handbook of Alcohol Dependence and Problems.* Sussex, England: John Wiley & Sons, Ltd; 2001.

83. Brownson-Smith R, Orange S, Cresti N, Hunt K, Saxton J, Temesi J. Effect of exercise before and/or during taxane-containing chemotherapy treatment on chemotherapy-induced peripheral neuropathy symptoms in women with breast cancer: systematic review and meta-analysis. *Journal of Cancer Survivorship.* 2023.

84. MacDonald A, Chafranskaia A, Lopez C, et al. CaRE @ Home: Pilot Study of an Online Multidimensional Cancer Rehabilitation and Exercise Program for Cancer Survivors. *Journal of Clinical Medicine.* 2020;9(10).

85. Trinh L, Arbour-Nicitopoulos K, Sabiston C, et al. RiseTx: testing the feasibility of a web application for reducing sedentary behavior among prostate cancer survivors receiving androgen deprivation therapy. *International Journal of Behavioral Nutrition and Physical Activity* 2018;15.

86. Brick R, Padgett L, Jones J, et al. The influence of telehealth-based cancer rehabilitation interventions on disability: a systematic review. *Journal of Cancer Survivorship.* 2022.

87. Sattar S, Papadopoulos E, Smith G, et al. State of research, feasibility, safety, acceptability, and outcomes examined on remotely delivered exercises using technology for older adult with cancer: a scoping review. *J Cancer Surviv.* 2023.

88. Winters-Stone K, Boisvert C, Li F, et al. Delivering exercise medicine to cancer survivors: has COVID-19 shifted the landscape for how and who can be reached with supervised group exercise?. . *Support Care Cancer.* 2022;30:1903-1906.

89. Myers S, Weller S, Schwartz S, et al. Feasibility of a Supervised Virtual Exercise Program for Women on Hormone Therapy for Breast Cancer. . *Translational Journal of the American College of Sports Medicine.* 2022;7.

90. Bland K, Kirkham A, Bovard J, et al. Effect of Exercise on Taxane Chemotherapy-Induced Peripheral Neuropathy in Women With Breast Cancer: A Randomized Controlled Trial. . *Clin Breast Cancer.* 2019;19(6):411-422.

91. Alfano CM, Ganz PA, Rowland JH, Hahn EE. Cancer Survivorship and Cancer Rehabilitation: Revitalizing the Link. . *Journal of Clinical Oncology* 2012;30 (9 ):904-906.

92. Eldridge S, Lancaster G, Campbell M, et al. Defining Feasibility and Pilot Studies in Preparation for Randomised Controlled Trials: Development of a Conceptual Framework. . *PLoS One.* 2016;11(3).

93. Eldridge S, Chan C, Campbell M, et al. CONSORT 2010 statement: extension to randomised pilot and feasibility trials. *BMJ.* 2016;355.

94. Basch E, Reeve B, Mitchell S, et al. Development of the National Cancer Institute’s patient-reported outcomes version of the common terminology criteria for adverse events (PRO-CTCAE). *J Natl Cancer Inst.* 2014;106(9).

95. Knoerl R, Mazzola E, Mitchell S, et al. Measurement properties of brief neuropathy screening items in cancer patients receiving taxanes, platinums, or proteasome inhibitors. . *J Patient Rep Outcomes.* 2021;5(1).

96. Bouhassira D, Attal N, Alchaar H, et al. Comparison of pain syndromes associated with nervous or somatic lesions and development of a new neuropathic pain diagnostic questionnaire (DN4). . *Pain.* 2005;114((1-2)):29-36.

97. Carroll KM. Enhancing Retention in Clinical Trials of Psychosocial Treatments: Practical Strategies. In: Onken LS, Blaine J, Boren J, eds. *Beyond the Therapeutic Alliance: Keeping the Drug-Dependent Individual in Treatment*: NIH Publication; 1997.

98. Chan A, Tetzlaff J, Gøtzsche P, et al. SPIRIT 2013 explanation and elaboration: guidance for protocols of clinical trials. . *BMJ.* 2013;346.

99. Michie S, Richardson M, Johnston M, et al. The behavior change technique taxonomy (v1) of 93 hierarchically clustered techniques: building an international consensus for the reporting of behavior change interventions. *Annals of Behavioral Medicine.* 2013;46(1):81-95.

100. Singh B, Zopf E, Howden E. Effect and feasibility of wearable physical activity trackers and pedometers for increasing physical activity and improving health outcomes in cancer survivors: A systematic review and meta-analysis. *J Sport Health Sci.* 2022;11(2):184-193.

101. Santarossa S, Kane D, Senn C, Woodruff S. Exploring the Role of In-Person Components for Online Health Behavior Change Interventions: Can a Digital Person-to-Person Component Suffice? . *J Med Internet Res.* 2018;20:e144.

102. Mohr D, Cuijpers P, Lehman K. Supportive accountability: a model for providing human support to enhance adherence to eHealth interventions. *J Med Internet Res.* 2011;13:e30.

103. Rollnick S, Miller W, Butler C, Aloia M. Motivational Interviewing in Health Care: Helping Patients Change Behavior. . *COPD: Journal of Chronic Obstructive Pulmonary Disease.* 2008;5:203-203.

104. Elley C, Kerse N, Arroll B, Robinson E. Effectiveness of counselling patients on physical activity in general practice: cluster randomised controlled trial. *BMJ.* 2003;326(7393):793.

105. Erlingsson C, Brysiewicz P. A hands-on guide to doing content analysis. *Afr J Emerg Med.* 2017;7(3):93-99.

106. MacQueen K, McLellan E, Kay K, Milstein B. Codebook Development for Team-Based Qualitative Analysis. *CAM Journal.* 1998;10(2):31-36.

107. Colorafi K, Evans B. Qualitative Descriptive Methods in Health Science Research. *HERD.* 2016;9(4):16-25.

108. Tong A, Sainsbury P, Craig J. Consolidated criteria for reporting qualitative research (COREQ): a 32-item checklist for interviews and focus groups. *Int J Qual Health Care.* 2007;19(6):349-357.

109. Hsieh H, Shannon S. Three approaches to qualitative content analysis. . *Qual Health Res.* 2005;15(9):1277-1288.

110. Jensen M, Karoly P, Braver S. The measurement of clinical pain intensity: a comparison of six methods. . *Pain.* 1986;27:117-126.

111. Farrar J, Young Jr J, LaMoreaux L, Werth J, Poole R. Clinical importance of changes in chronic pain intensity measured on an 11-point numerical pain rating scale. . *Pain.* 2001;94(2):149-158.

112. Postma T, Aaronson N, Heimans J, et al. The development of an EORTC quality of life questionnaire to assess chemotherapy-induced peripheral neuropathy: the QLQ-CIPN20. . *Eur J Cancer.* 2005;41(8):1135-1139.

113. Binda D, Vanhoutte E, Cavaletti G, et al. Rasch-built Overall Disability Scale for patients with chemotherapy-induced peripheral neuropathy (CIPN-R-ODS). *Eur J Cancer.* 2013;49(13):2910-2918.

114. Aaronson N, Ahmedzai S, Bergman B, et al. The European Organization for Research and Treatment of Cancer QLQ-C30: a quality-of-life instrument for use in international clinical trials in oncology. *J Natl Cancer Inst.* 1993;85(5):365-376.

115. Lewis M, Bromley K, Sutton C, McCray G, Myers H, Lancaster G. Determining sample size for progression criteria for pragmatic pilot RCTs: the hypothesis test strikes back! . *Pilot Feasibility Stud.* 2021;7(1).

116. Lancaster G, Dodd S, Williamson P. Design and analysis of pilot studies: recommendations for good practice. *Journal of evaluation in clinical practice.* 2004;**10**(2):307-312.

117. Sim J, Lewis M. The size of a pilot study for a clinical trial should be calculated in relation to considerations of precision and efficiency. *J Clin Epidemiol.* 2012;65(3):301-308.

118. Farrar J, Berlin J, Strom B. Clinically important changes in acute pain outcome measures: a validation study. . *J Pain Symptom Manage.* 2003;25(5):406-411.

119. Fereday J, Muir-Cochrane E. Demonstrating Rigor Using Thematic Analysis: A Hybrid Approach of Inductive and Deductive Coding and Theme Development. . *Int J Qual Methods.* 2006;5:80-92.

120. Thompson B. What future quantitative social science research could look like: Confidence intervals for effect sizes. *Educational Researcher.* 2002;31(3):25-32.

121. Hedges L, Olkin I. *Statistical Methods for Meta-analysis, Chapter 5.* San Diego: Academic Press; 1985.

122. Schmitz K, Holtzman J, Courneya KS, Masse L, Duval S, Kane R. Controlled physical activity trials in cancer survivors: A systematic review and meta-analysis. *Cancer epidemiology, biomarkers & prevention.* 2005;14(7):1588-1595.

#

| **Dimensions** | **Outcome assessment** | **Success Criteria** |  |  |  |
| --- | --- | --- | --- | --- | --- |
| **Primary Outcomes** |  |  |  |  |  |
| Feasibility |  |  |  |  |  |
| -          Accrual rate | Number of participants recruited monthly |  |  |  |  |
| -          Eligibility rate | Number (%) of recruited participants meeting inclusion criteria | >4 participants/month |  |  |  |
| -          Retention rate | Number (%) of randomized participants assessed at T2 and T3 | >70% of screened participants |  |  |  |
| -          Adherence | Health coaching call attendance: attends 9/10 calls | >70% of screened participants |  |  |  |
|  | Fitbit Usage: wears device for a minimum of 100min 9/10 weeks | >70% of screened participants |  |  |  |
|  | Self-reported completion of weekly exercise plan: completes 9/10 weeks | >70% of screened participants |  |  |  |
|  |  |  |  |  |  |
| Safety | CTCAE v 5 at assessments and during intervention | No Serious (Grade 3 and above) CTCAE |  |  |  |
|  |  |  |  |  |  |
| Acceptability | Surveys for all study participants | qualitative, not pre-defined |  |  |  |
|  | Semi-structured qualitative interview will explore participants experience in EX-CIPN (INT only) | qualitative, not pre-defined |  |  |  |
|  |  |  |  |  |  |
| Data completion rates | Number (%) of assessed participants reporting each outcome at T2 and T3 |  |  |  |  |
| **Secondary Outcomes** |  | **not applicable** |  |  |  |
| Pain (Primary clinical outcome) | Numeric Pain Rating Scale |  |  |  |  |
|  |  |  |  |  |  |
| CIPN Symptom severity | EORTC CIPN-20 |  |  |  |  |
|  |  |  |  |  |  |
| CIPN-related Disability | CIPN-RODS |  |  |  |  |
|  |  |  |  |  |  |
| Quality of Life | EORTC-QLCC30 |  |  |  |  |
|  |  |  |  |  |  |
| Physiological factors |  |  |  |  |  |
| -          Upper body strength | Grip dynamometer |  |  |  |  |
| -          Lower body strength | Sit to Stand Test |  |  |  |  |
| -          Balance | Berg Balance Test |  |  |  |  |
| -          Fitness | 6MWT |  |  |  |  |
| -          Gait speed | Gait speed test |  |  |  |  |
|  |  |  |  |  |  |

#

# Analysis of Secondary Outcomes

The following mixed effects model will be fit to estimate the outcome of participant $i$ at time $t$ the secondary outcomes:

$$y_{it}=\beta_{0}+u_{0i}+\beta_{1}T_{2i}+\beta_{2}T_{3i}+\beta_{3}T_{2i}x_{i}+\beta_{4}T_{3i}x_{i}$$

Where:

- $\beta_{0}$ is the average baseline ($T_{1}$) value and is assumed to be equal between groups due to randomization
- $u_{io}$ is a random baseline effect and assumed to be normally distributed across participants
- $T_{2i}$ and $T_{3i}$ are dummy-coded time indicators to enable estimation of the treatment effect after intervention ($T_{2}$) and at follow-up ($T_{3}$), respectively.
- $x_{i}$ is the treatment allocation of participant $i$
- $\beta_{1}$ and $\beta_{2}$ are the time effects evaluated at $T_{2}$ and $T_{3}$, respectively
- $\beta_{3}$ and $\beta_{4}$ are the intervention effects evaluated at $T_{2}$ and $T_{3}$, respectively

Data Structure:

| ID | Time | Group | x | T2 | T3 |
| --- | --- | --- | --- | --- | --- |
| 1 | T1 | Control | 0 | 0 | 0 |
| 1 | T2 | Control | 0 | 1 | 0 |
| 1 | T3 | Control | 0 | 0 | 1 |
| 2 | T1 | Intervention | 1 | 0 | 0 |
| 2 | T2 | Intervention | 1 | 1 | 0 |
| 2 | T3 | Intervention | 1 | 0 | 1 |

**Missing Data**

All observed data can be included in a mixed effects model, participants only observed at one or two timepoints will still contribute to the model estimates. No data will be imputed.
